# Associations between precipitation, temperature, and malaria prevalence in children under 5 in Mali

**DOI:** 10.1371/journal.pone.0342127

**Published:** 2026-02-20

**Authors:** Niklas J.T. Hayden, Joey Syer, Stephanie K. Yanow, Shelby S. Yamamoto

**Affiliations:** 1 School of Public Health, University of Alberta, Edmonton, Alberta, Canada; 2 School of Public Health and Health Professions, University at Buffalo, Buffalo, New York, United States of America; 3 Department of Medical Microbiology and Immunology, University of Alberta, Edmonton, Alberta, Canada; Clinton Health Access Initiative, UNITED STATES OF AMERICA

## Abstract

Malaria is prevalent in Mali and children under 5 are more vulnerable. Temperature and precipitation can affect vector density and parasite development, impacting malaria transmission. This exploratory study aimed to investigate potential associations between changes in precipitation and temperature and prevalence of malaria infection in children under 5 in Mali. A cross-sectional study was conducted using data from the Mali Demographic and Health Surveys and Malaria Indicators Surveys conducted in 2012/13, 2015, 2018, and 2021. We examined malaria prevalence diagnosed by rapid diagnostic tests (RDT) in children ages 6–59 months. Exposures included precipitation and temperature (minimum, maximum, average). Three monthly lags were created for each exposure. Multilevel modelling was conducted to assess the relationships between exposures and malaria for each lag in a pooled analysis of the survey years. With a three month lag, precipitation was statistically significantly positively associated with the odds of RDT-diagnosed malaria in children ages 6–59 months (Odds ratio (OR) [95% CI], 1.005 [1.002, 1.009]) and minimum, maximum, and average temperature were statistically significantly negatively associated with the odds of malaria (Minimum temperature = 0.761 [0.621, 0.931], maximum temperature = 0.871 [0.791, 0.958], average temperature = 0.822 [0.718, 0.942]). With a two month lag, maximum temperature was statistically significantly negatively associated with the odds of malaria (0.863 [0.750, 0.992]). At a one month lag, minimum temperature was statistically significantly positively associated with the odds of malaria (1.110 [1.014, 1.216]). All other results were not statistically significant. Precipitation may be a risk factor for malaria infection in children under 5 in Mali. Temperature alone is likely not contributing to changes in the odds of malaria infection, particularly when considering precipitation. Future studies focusing on regional-specific differences are needed to fully understand the relationships.

## 1. Introduction

### 1.1. Malaria

In 2023, 263 million malaria cases were reported globally [1]. Cases of malaria were declining globally up to 2015, then increased during the 2019−2020 year, coinciding with the beginning of the COVID-19 pandemic [1]. Notably, there were 236 million cases recorded in 2019 and 247 million in 2020 [1]. The observed declines occurring until 2015 may be attributed in part to interventions such as long-lasting insecticidal nets (LLINs) and indoor residual spraying (IRS), and to the efficacy of antimalarials [2]. Malaria-attributed mortality has been decreasing globally since 2000; however, there was a marked increase recorded in 2020, with 567,000 deaths in 2019 compared to the 622,000 in 2020 [1]. In 2023, 597,000 mortalities were recorded, a decrease from the previous year [1].

The life cycle of malaria parasites takes place in the female *Anopheles* mosquito as well as in infected humans [3]. Malaria parasites are transmitted to the human host from an infected mosquito during a blood meal [3]. The *Plasmodium* parasites first reproduce in hepatocytes then progress to the blood stages, where symptoms occur primarily due to schizont rupture and the destruction of erythrocytes [3,4]. The life cycle is completed in the *Anopheles* mosquito after it feeds on an infected human [3]. Climate and weather variables, such as precipitation and temperature, can have an important impact on malaria transmission by affecting the parasite development and vector density [5].

### 1.2. Malaria, weather, and climate

Precipitation has the ability to both benefit and hinder malaria transmission [6]. Precipitation can create more breeding sites for the vector, which in turn promotes malaria transmission and incidence [5]. However, an overabundance of rain may flood breeding sites which can reduce vector density [6]. Yamba et al. (2023) observed that the range of acceptability for rainfall differed between four African regions, but depending on the region, the maximum monthly rainfall value that allows for malaria transmission was around 400 mm or 600 mm [6]. The minimum was about 80 mm [6].

The impacts of temperature on malaria transmission are also range-dependent [7]. In the optimum range of temperatures, parasite development may be promoted and this can benefit malaria transmission [5,6]. Temperature can also affect factors such as mosquito fitness and life cycle which can in turn affect transmission [8]. The optimal range for high malaria transmission may be between 20°C to 28°C(9). Outside the optimal range, temperature may reduce malaria transmission, with below 15°C-16°C and above 40°C limiting parasite development [6]. These ranges are dependent on the mortality model [7]. With both precipitation and temperature, the exposure indirectly affects prevalence via effects on the vector/parasite. Many factors can impact malaria transmission contemporaneously, including climate and weather variables and the development of insecticide and antimalarial resistance, pandemics, financial and socioeconomic barriers, behavior, and access to healthcare [2,5,9,10].

### 1.3. Malaria, weather, and climate in Mali

There is sufficient evidence to suggest that meteorological variables, such as precipitation and temperature, affect malaria transmission in Mali and Africa in the general population [5,10]. In 2023, 94% of malaria cases (246,000/263,000) and 95% of malaria-attributable deaths (569,000/597,000) occurred within the WHO African Region [1]. Mali had 3.1% of all malaria cases globally (~8,153/263,000) and 2.4% of malaria-attributable deaths (~14,328/597,000) [1]. There are nuances, however, in the effect that climate and weather variables have in different regions, including Mali, such as geographical location, environment, and human behaviors [10]. For example, in Kolia, Mali, malaria transmission is sustained outside the rainy season by irrigation that keeps water from flowing downstream in the area [10]. According to a previous scoping review, the impact of temperature and precipitation on the prevalence of malaria in children under 5 has not been studied solely in Mali [11].

### 1.4. At-risk population

Of the deaths in the WHO African Region in 2023, 76% were among children under the age of five (~432,440/569,000) [1]. This population is particularly vulnerable as they lack acquired immunity [12]. As a result, in comparison to other populations, children under five have an increased likelihood of developing severe malaria which may include severe anemia, hypoglycemia, and cerebral malaria [12]. Furthermore, children are more vulnerable to the effects of climate change due to a number of factors, including physiology and behavior [13]. Compared to adults, children have less effective heat adaptation capacity and they spend more time outside [13].

### 1.5. The problem and objectives

Malaria is a prevalent disease in Mali that affects individuals of all ages in impactful ways [1,2,4,12]. The link between weather and malaria plays an important role in both the transmission and incidence of the disease [5]. Both temperature and precipitation have the capacity to promote and demote the spread of malaria [6]. It is important to develop an understanding of how climate and weather may impact malaria over time to inform policy and adaptation strategies in every country affected by malaria [14]. As such, the objective of this exploratory study was to investigate potential associations between precipitation and temperature and prevalence of malaria infection in children under 5 in Mali, a country with a lack of evidence on this question [11].

## 2. Materials and methods

### 2.1. Study design, population, and location

A cross-sectional study was conducted using historical data from 2012/13, 2015, 2018, and 2021 in Mali. Data sources included the Mali Demographic and Health Surveys (DHS) and Malaria Indicators Surveys (MIS) conducted in 2012/13 (Standard DHS), 2015 (MIS), 2018 (Standard DHS), and 2021 (MIS) [15–18]. DHS surveys are conducted by the DHS Program to collect primary data on a variety of public health topics, including malaria [19,20]. The DHS uses different types of questionnaires, including a household questionnaire and individual questionnaires [20]. These standardized surveys are conducted approximately every five years [19]. The MIS is an additional survey that collects data on malaria indicators, largely concerning children under five and pregnant women, and is also conducted every five years [21,22]. The DHS Program is implemented by ICF via the United States Agency for International Development (USAID) [23]. All DHS procedures and questionnaires are reviewed and approved by ICF Institutional Review Board (IRB) [24]. The data was initially accessed for research purposes on November 12, 2023. There is no information that can identify individual participants during or after data collection. Exact geographical coordinates for the surveys are not provided as the DHS program randomly displaces GPS latitude and longitude positions in all surveys for confidentiality purposes [25]. This research received ethics approval from the University of Alberta (ID: Pro00131799).

The population of Mali in 2023 was 23,769,127 [26]. The region was skewed towards younger ages with 46.8% of the population between the ages of 0–14 years and the median age being 16.4 years [27,28]. The population was 46.2% urban, with a 4.57% annual rate of urbanization [28]. Mali had an average household size of 5.8 people [26]. Around 7 out of 10 households had improved sources of drinking water access, 55% had improved sanitation, and 49% had electricity [26]. Concerning education and literacy, 66% of women and 53% of men had no education, and 2% of women and 6% of men had above a secondary education [26]. For literacy, 28% of women were literate compared to 47% of men [26].

**Fig 1:**
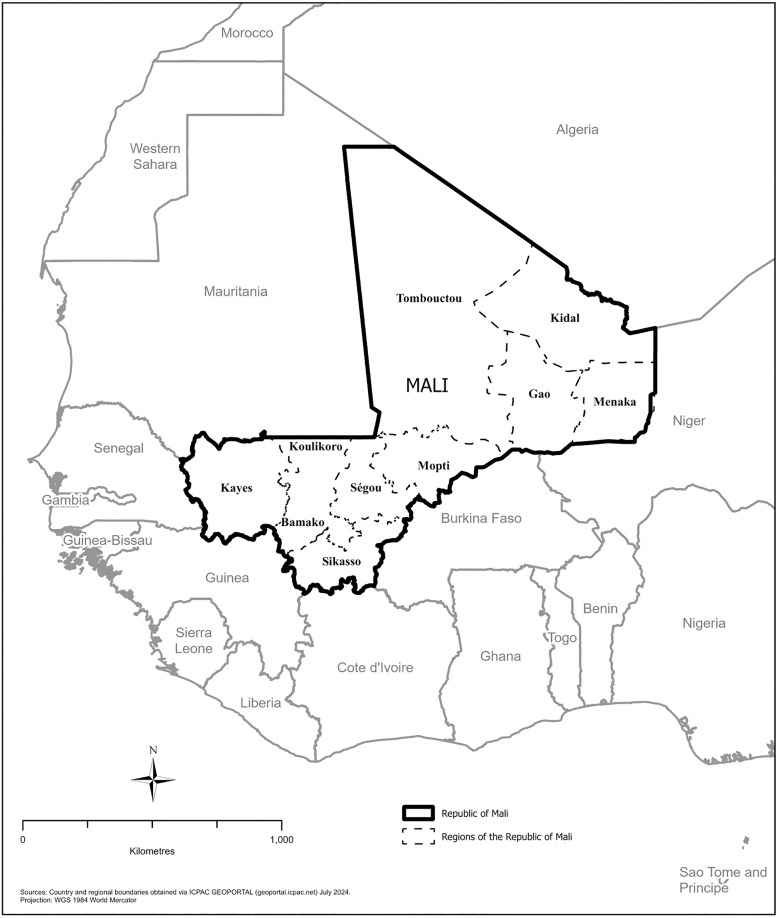
Regions of Mali.

In the DHS datasets, Mali is divided into 9 regions, including the capital of Bamako as its own region (Fig 1) [26]. The country is located in Western Africa, south of Algeria, and according to 2018 estimates, 34.1% of land is agricultural and 10.2% is forest area [28]. The vast majority of Malians live in the southern part of the country [28]. Mali’s climate is seasonal [29]. The rainy season is from May to October and peaks in August (average total precipitation of 109.75 mm across 1991–2020) [29,30]. The annual average mean surface air temperature in Mali was 29°C in 2012, 29.3°C in 2015, 29.36°C in 2018, and 29.81°C in 2021 [29]. Annual precipitation In Mali was 365.08 mm in 2012, 339.41 mm in 2015, 353.37 mm in 2018, and 321.85 mm in 2021 [29]. Temperature and precipitation also vary across the regions of Mali [29]. Malaria transmission typically peaks in October, at the end of the rainy season [31].

### 2.2. Outcome

The outcome of interest was malaria prevalence diagnosed by rapid diagnostic tests (RDT) in children ages 6–59 months. Malaria prevalence data were sourced from the Mali DHS and MIS. Participants were selected based on a stratified two-stage cluster design in which enumeration areas were based on census files and then a fixed number of households were selected by equal probability systematic sampling in each enumeration area [32,33]. The RDT sampling was done at the time of the interview with consent from parents [21]. Blood samples were immediately tested for anemia and malaria and results were given to participants during the interview [21]. The 2021, 2018, and 2015 surveys were conducted between August/September and November, overlapping with the rainy season [15–17,31]. The 2012/13 survey was conducted between November 2012 and February 2013 [18].

### 2.3. Exposure data

Exposures examined include the meteorological variables precipitation and temperature. Daily total precipitation (mm) data (2012/13, 2015, 2018, and 2021) were obtained from the Climate Hazards Group InfraRed Precipitation with Station (CHIRPS), which has a spatial resolution of 0.05° (~5.3 km) [34]. Monthly totals were calculated. Daily maximum and minimum temperature (°C) data were obtained from the Climate Prediction Center (CPC) Global Unified Temperature dataset from National Oceanic and Atmospheric Administration (NOAA) Physical Sciences Laboratory. Average temperature (°C) was generated by taking the average of the daily maximum and minimum temperatures. Monthly averages were used. Precipitation and temperature were spatially linked to DHS clusters using ArcGIS Pro. DHS surveys were conducted in different months, with some overlap between the years. Three lags were created for each exposure based on the month that any one individual was surveyed [35]. For example, a child surveyed in September would include temperature and precipitation exposures from June, July, August, and September. CHIRPS was created by the University of California, Santa Barbara Climate Hazards Center (CHC) in collaboration with the United States Geological Survey (USGS) Earth Resources Observation and Science (EROS) Center to provide complete data in regions where rain-gauge stations are not consistently placed [34]. The National Oceanic and Atmospheric Administration (NOAA) is a bureau within the United States Department of Commerce [36].

### 2.4. Covariates

DHS variables selected as relevant covariates are listed and defined in Table 1. Household-level variables included mother’s highest education level, type of place of residence, household electricity, main floor material, main roof material, main wall material, wealth index combined, mean cluster altitude (m), and the variable indicating whether the dwelling had been sprayed with insecticide in the last 12 months. The rest of the variables were treated as individual-level variables.

**Table 1:** Summary of the health outcome and characteristics for 2021, 2018, 2015, and 2012/13.

Population characteristics	2012/13 total population n (%)	2015 total population n (%)	2018 total population n (%)	2021 total population n (%)	Pooled total population n (%)
Sample size	N = 4517	N = 6929	N = 4287	N = 8571	N = 24304
**Health outcome**					
Malaria RDT					
Negative	2522 (55.8)	4777 (68.9)	3615 (84.3)	7037 (82.1)	17951 (73.9)
Positive	1995 (44.2)	2152 (31.1)	672 (15.7)	1534 (17.9)	6353 (26.1)
**Categorical characteristics**					
Sex					
Male	2311 (51.2)	3542 (51.1)	2200 (51.3)	4392 (51.2)	12445 (51.2)
Female	2206 (48.8)	3387 (48.9)	2087 (48.7)	4179 (48.8)	11859 (48.8)
Age in months (categorized)					
6-11	451 (10.0)	765 (11.0)	438 (10.2)	808 (9.4)	2462 (10.1)
12-23	938 (20.8)	1481 (21.4)	948 (22.1)	1746 (20.4)	5113 (21.0)
24-35	1000 (22.1)	1517 (21.9)	934 (21.8)	1955 (22.8)	5406 (22.2)
36-47	1099 (24.3)	1578 (22.8)	1012 (23.6)	2028 (23.7)	5717 (23.5)
48-59	1029 (22.8)	1588 (22.9)	955 (22.3)	2034 (23.7)	5606 (23.1)
Region					
Kayes	731 (16.2)	1274 (18.4)	548 (12.8)	1442 (16.8)	3995 (16.4)
Koulikoro	859 (19.0)	1348 (19.5)	570 (13.3)	1460 (17.0)	4237 (17.4)
Sikasso	810 (17.9)	1252 (18.1)	683 (15.9)	1442 (16.8)	4187 (17.2)
Segou	855 (18.9)	1225 (17.7)	538 (12.6)	1159 (13.5)	3777 (15.5)
Mopti	697 (15.4)	1013 (14.6)	351 (8.2)	690 (8.1)	2751 (11.3)
Tombouctou	–	–	479 (11.2)	614 (7.2)	1093 (4.5)
Gao	–	–	328 (7.7)	584 (6.8)	912 (3.8)
Kidal	–	–	276 (6.4)	390 (4.6)	666 (2.7)
Bamako	565 (12.5)	817 (11.8)	514 (12.0)	790 (9.2)	2686 (11.1)
Type of place of residence					
Urban	1057 (23.4)	1371 (19.8)	1139 (26.6)	1809 (21.1)	5376 (22.1)
Rural	3460 (76.6)	5558 (80.2)	3148 (73.4)	6762 (78.9)	18928 (77.9)
Cluster altitude in meters (categorized)^1^					
1^st^ quintile	878 (19.4)	1353 (19.5)	852 (19.9)	1615 (18.8)	4758 (19.6)
2^nd^ quintile	938 (20.8)	1414 (20.4)	802 (18.7)	1771 (20.7)	4867 (20.0)
3^rd^ quintile	902 (20.0)	1278 (18.4)	894 (20.9)	1713 (20.0)	4947 (20.4)
4^th^ quintile	866 (19.2)	1488 (21.5)	864 (20.2)	1703 (19.9)	4843 (19.9)
5^th^ quintile	933 (20.7)	1396 (20.2)	875 (20.4)	1769 (20.6)	4889 (20.1)
Has electricity					
No	3346 (74.1)	4633 (66.9)	2320 (54.1)	6287 (73.4)	16586 (68.2)
Yes	1171 (25.9)	2296 (33.1)	1967 (45.9)	2284 (26.7)	7718 (31.8)
Main floor material^2^					
Natural/other floor	3286 (72.8)	4774 (68.9)	2678 (62.5)	4647 (54.2)	15385 (63.3)
Rudimentary floor	70 (1.6)	–	81 (1.9)	72 (0.8)	223 (0.9)
Finished floor	1161 (25.7)	2155 (31.1)	1528 (35.6)	3852 (44.9)	8696 (35.8)
Main roof material^3^					
Natural/other roof	1454 (32.2)	2805 (40.5)	1084 (25.3)	2430 (28.4)	7773 (32.0)
Rudimentary roof	678 (15.0)	–	668 (15.6)	442 (5.2)	1788 (7.4)
Finished roof	2385 (52.8)	4124 (59.5)	2535 (59.1)	5699 (66.5)	14743 (60.7)
Main wall material^4^					
Natural/other walls	3022 (66.9)	3848 (55.5)	1543 (36.0)	3254 (38.0)	11667 (48.0)
Rudimentary walls	416 (9.2)	824 (11.9)	939 (21.9)	1034 (12.1)	3213 (13.2)
Finished walls	1079 (23.9)	2257 (32.6)	1805 (42.1)	4283 (50.0)	9424 (38.8)
Mother’s highest education level					
No education	3494 (82.7)	4926 (78.1)	2914 (74.3)	5439 (70.2)	16773 (75.6)
Primary	399 (9.4)	730 (11.6)	489 (12.5)	1087 (14.0)	2705 (12.2)
Secondary	303 (7.2)	592 (9.4)	472 (12.0)	1114 (14.4)	2481 (11.2)
Higher	29 (0.7)	57 (0.9)	48 (1.2)	105 (1.4)	239 (1.1)
Slept under a mosquito bed net the previous night					
Did not sleep under a net	1269 (28.1)	1896 (27.4)	985 (23.0)	2315 (27.0)	6465 (26.6)
Slept under only untreated net	42 (0.9)	68 (1.0)	128 (3.0)	212 (2.5)	450 (1.9)
Slept under treated (ITN) net	3206 (71.0)	4965 (71.7)	3174 (74.0)	6044 (70.5)	17389 (71.6)
Wealth index^5^					
Poorest	919 (20.4)	1402 (20.2)	905 (21.1)	1752 (20.4)	4978 (20.5)
Poorer	941 (20.8)	1595 (23.0)	907 (21.2)	1894 (22.1)	5337 (22.0)
Middle	907 (20.1)	1505 (21.7)	863 (20.1)	1837 (21.4)	5112 (21.0)
Richer	848 (18.8)	1338 (19.3)	894 (20.9)	1725 (20.1)	4805 (19.8)
Richest	902 (20.0)	1089 (15.7)	718 (16.8)	1363 (15.9)	4072 (16.8)
Medication taken for fever^6^					
No/don’t know if fever in last 2 weeks/ Nothing taken	3823 (92.3)	5190 (81.9)	3382 (90.7)	–	–
Painkiller, antibiotic, or other taken	216 (5.2)	596 (9.4)	230 (6.2)	–	–
Antimalarial taken	101 (2.4)	554 (8.7)	116 (3.1)	–	–
Received drugs to prevent malaria this month or last					
No/don’t know	–	–	–	3384 (44.1)	–
Yes	–	–	–	4293 (55.9)	–
Given medication to prevent malaria in applicable year^7^					
Nothing taken/don’t know	–	3372 (53.5)	1510 (40.5)	–	–
Traditional medication or other taken	–	126 (2.0)	59 (1.6)	–	–
Antimalarial taken	–	2807 (44.5)	2159 (57.9)	–	–
Has dwelling been sprayed in last 12 months?					
No/don’t know	4134 (91.5)	6488 (93.6)	–	–	–
Yes	383 (8.5)	441 (6.4)	–	–	–
**Continuous characteristics**	n, mean/median, standard deviation/ interquartile range	n, mean/median, standard deviation/ interquartile range	n, mean/median, standard deviation/ interquartile range	n, mean/median, standard deviation/ interquartile range	n, mean/median, standard deviation/ interquartile range
Mean hemoglobin level adjusted for altitude (g/dl – 1 decimal)^8^	n = 4517 94.2 (17.9)	n = 6925 92.7 (16.7)	n = 4279 96.4 (15.8)	n = 8570 98.6 (14.8)	n = 24291 95.7 (16.3)
Medan Body Mass Index (BMI)(kg/m^2^)	n = 4473 15.5 (14.3-16.4)	–	n = 4284 15.2 (14.3-16.2)	–	–

^1^Quintiles for 2021 were <268, 268 to <293, 293 to <334, 334 to <365, and ≥365. Quintiles for 2018 were <269, 269 to <292, 292 to <331, 331 to <361, and ≥361. Quintiles for 2015 were <274, 274 to <297, 297 to <329, 329 to <354, and ≥354. Quintiles for 2012/13 were <279, 279 to <306, 306 to <331, 331 to <357, and ≥357.

^2^Natural/other floor could include earth/sand, dung, and/or other [37,38]. Rudimentary floor could include wood planks and/or palm/bamboo [37,38]. Finished floor could include parquet or polished wood, vinyl or asphalt strips, ceramic tiles, cement, and/or carpet [37,38]. See S2 Tables for tabular presentation.

^3^Natural/other roof could include no roof, thatch/palm leaf, sod, and/or other [37,38]. Rudimentary roof could include rustic mat, palm/bamboo, wood planks, cardboard, and/or tarpaulin/plastic [37,38]. Finished roof could include metal, wood, zinc/calamine/cement fiber, roof/ceramic tiles, cement, and/or roofing shingles [37,38]. See S2 Tables for tabular presentation.

^4^Natural/other walls could include no walls, bamboo/cane/palm/trunks, dirt, and/or other [37,38]. Rudimentary walls could include bamboo with mud, stone with mud, uncovered adobe, plywood, cardboard, and/or reused wood [37,38]. Finished walls could include cement, stone with lime/cement, bricks, cement blocks, covered adobe, and/or wood planks/shingles [37,38]. See S2 Tables for tabular presentation.

^5^The wealth index uses data on each household’s ownership of items such as televisions and bicycles; materials used for housing construction; and types of water access and sanitation facilities to place households in a continuous scale of relative wealth [39]. Households are placed into quintiles for the purpose of comparison and principal components analysis is used to create the index [39].

^6^Antimalarial taken means at least an antimalarial was taken but something else could have been taken in addition. Painkiller, antibiotic, or other taken means one of these was taken but an antimalarial was not taken. Nothing taken/don’t know means nothing was taken or they don’t know if or what was taken. Antimalarials could include fansidar, Sulfadoxine pyrimethamine/fansidar, chloroquine, amodiaquine, quinine, quinine pill, quinine injection/iv, combination with artemisinin, artesunate rectal, artesunate injection/iv, fansidar and amodiaquine (combined), and/or other antimalarial. Antibiotics could include antibiotic pill/syrup, antibiotic injection, and/or kunbileni. Painkillers could include aspirin, aspirin/paracetamol/panadol, acetaminophen/paracetamol/panadol, and/or ibuprofen. See S2 Tables for tabular presentation.

^7^Antimalarial taken means at least an antimalarial was taken but something else could have been taken in addition. Traditional medication or other taken means one of these was taken but an antimalarial was not taken. Nothing taken/don’t know means nothing was taken or they don’t know if or what was taken. Antimalarials could include sulfadoxine pyrimethamine/fansidar and amodiaquine (both bag and package), fansidar and amodiaquine (both box and package), fansidar alone, sulfadoxine pyrimethamine/fansidar alone, amodiaquine alone, ACT, chloroquine, quinine, and/or other antimalarials. Traditional medicine could include decoction/plant/root juice and/or other traditional medicine. See S2 Tables for tabular presentation.

^8^Severe anemia = below 7.0 g/dl, moderate = between 7.1g/dl and 9.9g/dl, mild = between 10.0 g/dl and 10.9 g/dl [39].

### 2.5. Statistical analysis

**Fig 2:**
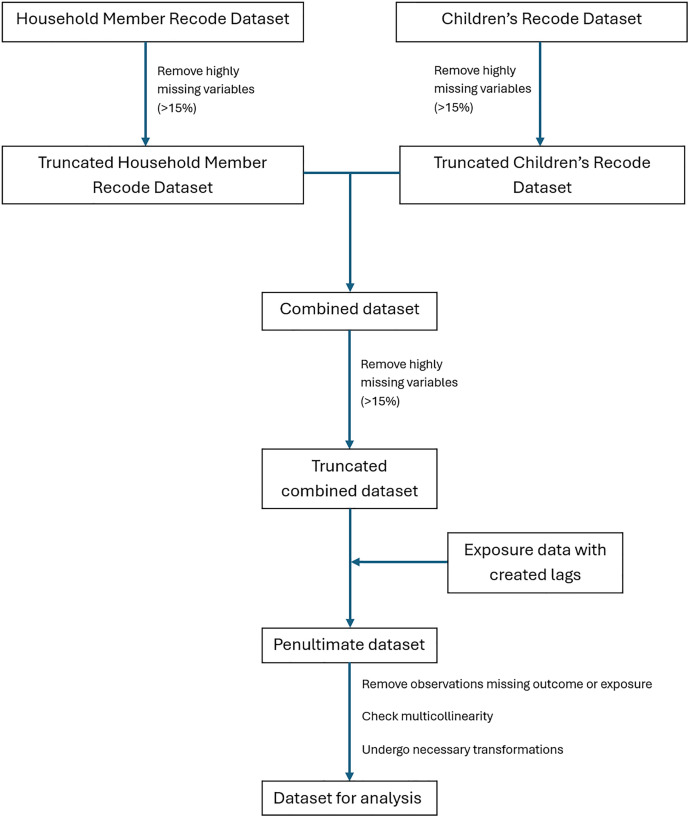
Data management flowchart.

Individual-level (covariates and outcome), household-level (covariates), and cluster-level (exposures) data were used to analyze the associations between temperature and precipitation exposures and malaria diagnosis in children under 5 in Mali. Different DHS datasets (Household Member Recode and Children’s Recode datasets) were merged by using multiple variables in the dataset to uniquely identify each child (using the child’s line number in the household, the cluster number, the household number, and the respondent’s (mother’s) line number). These data, including the covariate and outcome data, were subsequently merged with the exposure data using the cluster number and date measured (month). Missing data were checked individually in the Household Member Recode and Children’s Recode datasets, as well as in the combined dataset, to ensure that variables did not contain a high degree of missingness (>15% missingness). Observations missing either the outcome or exposure were excluded. The 2018 dataset was the only one to have high degrees of missingness, but missingness was less than 15% after dropping observations which did not have a response for malaria RDT. Transformations were undertaken for continuous variables using skewness and kurtosis values. Body mass index was logarithmically transformed, age and altitude were categorized, all other continuous variables were assessed as normal. Model variables were selected *a priori*. Multicollinearity was checked; anemia level and hemoglobin level adjusted for altitude were highly multicollinear, as was slept under a mosquito bed net the previous night and person slept under a long-lasting insecticidal net (LLIN). Hemoglobin level adjusted for altitude and slept under a mosquito bed net the previous night were kept in the analysis as they included more detail. Anemia level and person slept under a long-lasting insecticidal net (LLIN) were excluded (Fig 2).

Variables were grouped into four categories for entry into the models: demographics (sex, type of place of residence, mother’s highest education level, age, and cluster altitude in meters), economics (household electricity, main floor material, main roof material, main wall material, and wealth index combined), biological (hemoglobin level adjusted for altitude and BMI), and interventions (slept under a mosquito bed net the previous night, medication taken for fever, received drugs to prevent malaria current month or last, given medication to prevent malaria in applicable year, and dwelling sprayed in last 12 months). This was done so that covariates could be entered into the model sequentially to avoid the potential for overfitting.

Logistic regression was conducted to assess univariate and multivariable relationships between exposures (minimum temperature, maximum temperature, average temperature, precipitation) and malaria for each lag (0, 1, 2, 3) in a pooled analysis of all of the years (2021, 2018, 2015, 2012/13). Two-level multilevel modeling was used where the first level was the individual and the second level was the cluster. Only covariates which were available in all years were included in the analysis. Grouped covariates were sequentially added to the models (final model adjusted for all covariates). A sensitivity analysis was performed to ensure that the exclusion of selected covariates (medication taken for fever, given medication to prevent malaria in applicable year, body mass index (BMI), has dwelling been sprayed in last 12 months, received drugs to prevent malaria this month or last) did not statistically significantly impact the results of the model (S3 Tables). A pooled meta-analysis was attempted, though not successful due to high heterogeneity between the years. Year was included as a covariate in the pooled analyses to adjust for this heterogeneity. Additionally, multilevel logistic regression models were developed for all years individually (S4 Tables).

Variable categories were added sequentially: univariate (model 1), adding demographics (model 2), adding economics (model 3), adding biological (model 4), and adding interventions (model 5/final model). Effect modification by age and sex was assessed in the pooled analysis and individual year models (S5 Tables). All models included multilevel weights [40]. All analyses were completed in STATA MP/18.

## 3. Results

### 3.1. Descriptive summary

#### 3.1.1. Population.

Descriptive statistics were summarized for the pooled results (Table 1). The sample size for the total population of children aged 6–59 months was 24,304. Age was categorized with 10.1% of children in the 6–11 months group and 21.0–23.5% for each of the other age categories. The percentage of males (51.2%) to females (48.8%) was close to even. The sample was predominantly rural (77.9%) with a smaller urban sample (22.1%). Most individuals did not have electricity (68.2%). Mothers usually reported having no formal education (75.6%) and only 1.1% had a higher education. The wealth index and cluster altitude were divided into quintiles. Floor materials were mostly natural (63.3%), whereas roof material was mostly finished (60.7%). Wall material was 48.0% natural and 38.8% finished. Most children slept under an insecticide-treated net (ITN) the previous night (71.6%), while 26.6% did not sleep under a net at all. The mean hemoglobin level adjusted for altitude (g/dL) was 95.7 with a standard deviation of 16.3. Population characteristics were also summarized for the yearly populations.

#### 3.1.2. Malaria.

The percentage of children who tested positive for malaria by RDT in the pooled population was 26.1% (6,353/24,304). For the yearly populations, the percentage of children who tested positive for malaria by RDT in 2012/13 was 44.2% (1,995/4,517). This number decreased in 2015 to 31.1% (2,152/6,929) and decreased again in 2018 to 15.7% (672/4,287), then was followed by a small increase (2.2%) in 2021 with 17.9% positive cases (1,534/8,571). A descriptive summary of malaria prevalence diagnosed by rapid diagnostic tests (RDT) in children ages 6–59 months is presented in Table 1.

#### 3.1.3. Exposures.

The median pooled precipitation level across Mali varied depending on the lag (Table 2). The smallest median is with a zero month lag (43.7 mm); this is opposed to the largest median which is with a two month lag (169.6 mm). The highest median temperature was 33.9°C with a zero month lag and the lowest median temperature was 22.9°C with the same lag. The highest median for average temperature was 29.1°C with a three month lag and the lowest median for average temperature 28.0°C with a one month lag. Exposure descriptives have also been summarized for the yearly populations.

**Table 2:** Summary of the exposures for 2021, 2018, 2015, and 2012/13.

	2012/13 n, median, interquartile range	2015 n, median, interquartile range	2018 n, median, interquartile range	2021 n, median, interquartile range	Pooled n, median, interquartile range
Sample size	N = 4517	N = 6929	N = 4287	N = 8571	N = 24304
**Exposure (lag #)** ^**1**^					
Precipitation (lag 0)	4830, 0.3, 0.0-1.6	6989, 38.8, 1.0-93.3	4064, 147.6, 41.2-222.1	8634, 59.6, 37.2-137.0	23895, 43.7, 1.5-115.2
Precipitation (lag 1)	4830, 0.5, 0.0-6.1	6989, 170.5, 35.6-213.8	4064, 207.2, 120.0-243.3	8634, 184.2, 101.7-279.3	23895, 142.6, 31.9-220.4
Precipitation (lag 2)	4830, 21.4, 2.6-78.0	6989, 201.6, 164.2-228.7	4064, 155.2, 95.7-234.7	8634, 194.3, 115.8-253.6	23895, 169.6, 91.4-233.0
Precipitation (lag 3)	4830, 99.2, 52.9-175.9	6989, 188.8, 149.1-247.2	4064, 79.0, 32.9-140.5	8634, 106.8, 62.1-153.7	23895, 132.4, 70.2-189.3
Minimum temperature (lag 0)	4830, 18.5, 17.9-19.3	6989, 22.4, 20.7-23.5	4064, 23.6, 22.7-24.5	8634, 24.1, 22.8-25.2	23895, 22.9, 21.2-24.3
Minimum temperature (lag 1)	4830, 22.0, 18.8-23.2	6989, 23.3, 22.6-25.0	4064, 23.6, 22.7-25.0	8634, 23.7, 22.6-24.7	23895, 23.3, 22.4-24.5
Minimum temperature (lag 2)	4830, 23.4, 22.1-24.3	6989, 23.4, 23.0-24.0	4064, 24.5, 23.1-26.2	8634, 24.0, 23.2-25.8	23895, 23.7, 23.0-24.8
Minimum temperature (lag 3)	4830, 22.7, 22.2-23.3	6989, 24.0, 23.3-24.5	4064, 26.5, 24.5-27.6	8634, 25.3, 24.0-27.2	23895, 24.2, 23.3-26.0
Maximum temperature (lag 0)	4830, 29.5, 29.0-31.8	6989, 35.2, 33.2-36.2	4064, 32.3, 30.6-34.9	8634, 34.9, 33.3-36.6	23895, 33.9, 31.4-35.9
Maximum temperature (lag 1)	4830, 33.7, 29.9-34.9	6989, 31.9, 31.1-35.6	4064, 31.9, 30.9-34.5	8634, 32.7, 31.3-34.2	23895, 32.5, 31.0-34.9
Maximum temperature (lag 2)	4830, 34.3, 32.3-35.6	6989, 32.2, 31.1-32.5	4064, 34.0, 31.4-36.6	8634, 32.7, 31.3-35.2	23895, 32.5, 31.3-34.9
Maximum temperature (lag 3)	4830, 31.9, 30.5-34.1	6989, 32.7, 32.2-33.7	4064, 37.6, 34.3-40.2	8634, 36.0, 32.9-38.6	23895, 33.8, 32.2-37.0
Average temperature (lag 0)	4830, 24.0, 23.5-25.8	6989, 28.3, 27.7-29.2	4064, 27.8, 26.9-29.6	8634, 29.6, 28.1-30.9	23895, 28.2, 27.1-29.8
Average temperature (lag 1)	4830, 27.9, 24.3-29.0	6989, 27.7, 26.9-30.4	4064, 27.7, 26.8-29.6	8634, 28.2, 27.2-29.4	23895, 28.0, 26.7-29.5
Average temperature (lag 2)	4830, 28.6, 27.3-30.0	6989, 27.8, 27.1-28.2	4064, 29.3, 27.2-31.3	8634, 28.4, 27.3-30.4	23895, 28.2, 27.2-29.9
Average temperature (lag 3)	4830, 27.5, 26.4-28.4	6989, 28.3, 27.7-29.3	4064, 32.1, 29.4-34.3	8634, 30.7, 28.5-32.9	23895, 29.1, 27.8-31.7

^1^Precipitation is in millimeters and temperature exposures are in degrees Celsius. Temporal resolution is monthly.

### 3.2. Associations with exposures

#### 3.2.1. Precipitation.

Precipitation was statistically significantly associated with the odds of malaria with a three month lag, where a statistically significant risk factor result was observed for precipitation in every model (Table 3). The adjusted odds of being positive for malaria RDT in children ages 6–59 months was 1.005 times greater for every millimeter increase in total precipitation for the three month lag in the pooled models.

**Table 3:** Adjusted multilevel logistic regression model associations for every lag, pooled across 2021, 2018, 2015, and 2012/13, for every exposure variable with malaria prevalence diagnosed by RDT in children ages 6 to 59 months.

Exposure variable	Lag	Model 1 OR (95% CI)^1^	Model 2 OR (95% CI)^2^	Model 3 OR (95% CI)^3^	Model 4 OR (95% CI)^4^	Model 5 OR (95% CI)^5^
Precipitation	0	0.998 (0.995, 1.002)	0.999 (0.996, 1.002)	0.999 (0.996, 1.002)	0.999 (0.996, 1.002)	0.999 (0.996, 1.002)
	1	1.000 (0.997, 1.004)	1.001 (0.997, 1.004)	1.001 (0.997, 1.004)	1.001 (0.997, 1.004)	1.001 (0.997, 1.004)
	2	1.000 (0.997, 1.004)	1.001 (0.997, 1.005)	1.001 (0.998, 1.005)	1.001 (0.997, 1.005)	1.001 (0.997, 1.005)
	3	1.005 (1.001, 1.008)*	1.005 (1.002, 1.009)*	1.006 (1.002, 1.009)*	1.005 (1.002, 1.009)*	1.005 (1.002, 1.009)*
Minimum temperature	0	0.980 (0.906, 1.060)	0.979 (0.906, 1.058)	0.976 (0.905, 1.053)	0.972 (0.901, 1.048)	0.972 (0.902, 1.048)
	1	1.102 (0.989, 1.227)	1.120 (1.009, 1.244)*	1.106 (1.001, 1.222)*	1.110 (1.014, 1.215)*	1.110 (1.014, 1.216)*
	2	0.859 (0.727, 1.014)	0.861 (0.681, 1.088)	0.829 (0.660, 1.042)	0.844 (0.670, 1.064)	0.845 (0.670, 1.065)
	3	0.813 (0.691, 0.955)*	0.791 (0.640, 0.977)*	0.762 (0.620, 0.936)*	0.760 (0.620, 0.931)*	0.761 (0.621, 0.931)*
Maximum temperature	0	0.953 (0.875, 1.037)	1.018 (0.940, 1.103)	1.004 (0.928, 1.085)	0.997 (0.924, 1.076)	0.997 (0.924, 1.076)
	1	0.994 (0.908, 1.088)	1.007 (0.918, 1.103)	1.002 (0.918, 1.093)	1.010 (0.939, 1.087)	1.010 (0.938, 1.087)
	2	0.875 (0.776, 0.987)*	0.862 (0.753, 0.986)*	0.845 (0.740, 0.965)*	0.863 (0.750, 0.992)*	0.863 (0.750, 0.992)*
	3	0.883 (0.805, 0.970)*	0.880 (0.797, 0.971)*	0.866 (0.786, 0.954)*	0.870 (0.791, 0.957)*	0.871 (0.791, 0.958)*
Average temperature	0	0.953 (0.868, 1.046)	1.001 (0.916, 1.095)	0.990 (0.907, 1.080)	0.982 (0.901, 1.071)	0.982 (0.901, 1.071)
	1	1.029 (0.931, 1.138)	1.048 (0.948, 1.159)	1.039 (0.944, 1.144)	1.047 (0.965, 1.137)	1.047 (0.965, 1.137)
	2	0.856 (0.739, 0.991)*	0.842 (0.703, 1.007)	0.819 (0.687, 0.977)*	0.838 (0.698, 1.006)	0.838 (0.698, 1.007)
	3	0.849 (0.751, 0.959)*	0.839 (0.729, 0.965)*	0.819 (0.713, 0.939)*	0.822 (0.718, 0.941)*	0.822 (0.718, 0.942)*

* p < 0.05

^1^Adjusted for Year

^2^Adjusted for Year, Sex, Urban/rural, Mothers’ education, Age, Altitude

^3^Adjusted for Year, Sex, Urban/rural, Mothers’ education, Age, Altitude, Household electricity, Floor/Roof/Wall material, Wealth index

^4^Adjusted for Year, Sex, Urban/rural, Mothers’ education, Age, Altitude, Household electricity, Floor/Roof/Wall material, Wealth index, Hemoglobin level adjusted for altitude

^5^Adjusted for Year, Sex, Urban/rural, Mothers’ education, Age, Altitude, Household electricity, Floor/Roof/Wall material, Wealth index, Hemoglobin level adjusted for altitude, Slept under a mosquito bed net the previous night

#### 3.2.2. Minimum temperature.

Minimum temperature was statistically significantly associated with the odds of malaria with a one month and three month lag, where a statistically significant risk factor result was observed for the one month lag and a statistically significant protective result was observed for the three month lag (Table 3). The zero month and two month lags were not statistically significantly associated with the odds of malaria. The adjusted odds of testing positive by RDT in children ages 6–59 months was 1.110 times greater for every degree Celsius increase in average daily minimum temperature with a one month lag. The adjusted odds of being positive for malaria RDT in children ages 6–59 months was 0.761 times lower for every degree Celsius increase in average daily minimum temperature with a three month lag.

#### 3.2.3. Maximum temperature.

Maximum temperature was statistically significantly associated with the odds of malaria with a two month and three month lag, where a statistically significant protective result was observed for both lags (Table 3). The zero month and one month lags were not statistically significantly associated with the odds of malaria. The adjusted odds of being positive for malaria RDT in children ages 6–59 months was 0.863 times lower for every degree Celsius increase in average daily maximum temperature with a two month lag. The adjusted odds of being positive for malaria RDT in children ages 6–59 months was 0.871 times lower for every degree Celsius increase in average daily maximum temperature with a three month lag.

#### 3.2.4. Average temperature.

Average temperature was statistically significantly associated with the odds of malaria with a three month lag, where a statistically significant protective result was observed in every model (Table 3). The adjusted odds of testing positive by RDT in children ages 6–59 months was 0.822 times lower for every degree Celsius increase in average daily average temperature with a three month lag.

#### 3.2.5. Results summary.

The evidence suggests that the odds of testing positive for malaria by RDT for children ages 6–59 months in Mali may be impacted by precipitation and temperature, particularly at a three month lag between the exposure and the outcome. Not all exposures were related to the outcome in the same direction; whereas precipitation was a risk factor for a positive malaria RDT with a three month lag, all three temperature exposures were protective. However, while these environmental exposures can impact the odds of testing positive for malaria by RDT for children ages 6–59 months in Mali, it is dependent on the lag and may also be dependent on other factors not fully assessed, such as season [41]. A preliminary assessment accounting for seasonality suggested that season is not impactful on the final results.

Effect modification by age and sex was assessed (S5 Tables). There is insufficient evidence for effect modification by sex in the pooled results. There is evidence of effect modification by age with the precipitation and maximum temperature exposures at certain lags; however, the three month lag trends in the pooled analysis across age groups were consistent.

### 3.3. Sensitivity analysis

A sensitivity analysis was completed comparing the main pooled analysis with models including the covariates (medication taken for fever, given medication to prevent malaria in applicable year, body mass index (BMI), has dwelling been sprayed in last 12 months, received drugs to prevent malaria this month or last) excluded in the main analysis due to selected missingness across survey years. The models including these excluded variables were constructed by selectively pooling the years in which these covariates were available. These models followed similar trends to the main analysis suggesting that they did not significantly impact the main findings (S3 Tables). Data for each of the years were also analysed separately (S4 Tables) and did not generate the same results, though this was likely due to sample size limitations.

## 4. Discussion

Temperature and precipitation may affect the transmission and incidence of malaria, a disease which is prevalent in Mali and is impactful on human health [1,2,4,5,6,12]. The objective of this exploratory study was to investigate potential associations between changes in precipitation and temperature and prevalence of malaria infection in children under 5 in Mali to contribute to a body of literature which may inform policy and adaptation strategies in the country [14].

Results varied depending on the exposure and the number of lagged months. Precipitation associations were most consistent with a three month lag, indicating a slight increased risk of malaria. However, minimum, maximum, and average temperature were all statistically significantly protective with a three month lag period. The adjusted odds of testing positive for malaria by RDT in children ages 6–59 months was lower for every degree Celsius increase in the average daily temperature. Maximum temperature was also statistically significantly protective for every degree Celsius increase at a two month lag with a median temperature of 32.5°C. We observed the median temperature for a one month lag for minimum temperature (23.3°C) was associated with an increased odds of malaria. The sensitivity analysis found that the main pooled analysis and the models including the covariates excluded followed similar trends, suggesting that the excluded variables did not significantly impact the main findings (S3 Tables). Variables on medication, body mass index (BMI), and IRS should still be considered in future analysis given their public health significance.

Parasite development can be hindered in the mosquito at higher temperatures, though the temperatures at a three month lag were below 40°C, the hypothesized threshold [6,7]. In a study conducted in nine sub-Saharan African countries (Nigeria, Ethiopia, South Africa, Kenya, Uganda, Ghana, Mozambique, Zambia and Zimbabwe), an increase in temperature was associated with a decrease in malaria incidence rates, particularly in select countries with high annual average temperatures [42,43]. A similar trend might occur in Mali, which has an average temperature between 24°C in January and 35°C in May [29]. This inverse relationship may be due to the influence of other factors, such as humidity, variability in precipitation, and geographical factors [42]. Increased humidity has been associated with an increase in malaria transmission but was not assessed in the present study [42]. These results suggest that temperature alone may not be contributing to reducing malaria infection, particularly when combined with the precipitation findings.

Similar heterogeneity in results was reported across studies examining the link between precipitation and temperature and malaria infection in children under 5 in sub-Saharan Africa [11]. The most consistent finding in the present study across exposures was with a three month lag, suggesting that a three month lag was the most influential lag period. Lag periods are important because of the influence of the malaria transmission cycle, resulting in a delay between the exposures and their effect on prevalence [35]. The significance of the three month lag may also be influenced by the development time and survival of the vector, and the incubation period of the disease. It is possible that this delay had not elapsed in the earlier lags, meaning the exposure would not affect the outcome.

The results suggest that precipitation may be a risk factor for malaria infection in children under 5 in Mali, and that this risk is maintained even in the presence of countermeasures such as the use of mosquito bed nets. The effect size of precipitation, though relatively small, is still significant in terms of public health. Small effect sizes can be impactful in environmental contexts in which there is a high baseline rate of the health outcome, and many are subject to the exposure [44]. Similar results for precipitation and/or temperature were reported in other sub-Saharan African countries (e.g., Uganda, Burkina Faso, Kenya, Zambia, and Malawi), but the specifics of any one study is complex [35,45–49]. For example, in Uganda, Ssempiira et al. reported that malaria incidence in children under 5 was both positively associated and not significantly associated with rainfall depending on the level of rainfall while day land surface temperature was negatively associated and positively associated with malaria incidence depending on the temperature [35].

These findings suggest that the relationship between changes in precipitation and temperature and prevalence of malaria infection in children under 5 in Mali is complicated. This relationship is likely affected by factors beyond just environmental exposures. While similar results were reported in other countries, there is heterogeneity in examining the relationship between environmental exposures and malaria infection in children under 5 [11]. One potential explanation is that the specifics of any country, or region within that country, changes the context of malaria infection [6,7]. Different countries in sub-Saharan Africa have, to varying degrees, implemented improved access to healthcare and coverage for LLINs, seasonal malaria chemoprevention, and IRS [50]. Time children spend outside in the early evening or night or the health-seeking attitude of mothers can influence malaria incidence, which may be affected by weather and differ across regions [51,52]. Countries optimize the limited funding for malaria control programmes by relying on the context-specifics of settings/regions [50,53]. Future studies may benefit from validating the environmental exposures with local outcome data. Multiple studies may be needed to understand the full breadth of the relationships.

### 4.1. Limitations

This study has limitations which must be acknowledged. The sample size of any one survey was limited. While pooling of the years increased statistical power, the different times that the field work was conducted between these surveys may have increased heterogeneity [54]. The analysis was conducted on data from Mali as a whole without accounting for how this relationship may vary geographically across the country. Selected variables were omitted due to collinearity, which may have impacted the association between the outcome and the exposure, though sensitivity analyses were undertaken to test the consistency of the results. Potential confounders, such as mother’s occupation, time children spent outdoors, and the health-seeking attitude of the mother, were not included in the study. Due to the limitations of RDT, it is possible that lower level infections could have been missed [55]. Undetected cases of malaria are still affected by environmental exposures. RDT is also incapable of differentiating between untreated current infections and recently treated current infections [55]. Variables required assumptions, for example the use of the variable “slept under a mosquito bed net the previous night” assumes that previous night is representative of most/all nights. Generalizability is limited; the effect of these exposures varies across regions, as such the results of this study may not extend outside of Mali [11]. The DHS may suffer from volunteer and nonresponse bias wherein ill individuals may be more likely to participate than healthy individuals and respond to different questions. Exclusion bias, which is bias that occurs from the exclusion of a part of the population, occurred in the DHS surveys of Mali due to the purposeful exclusion of regions for security reasons.

## 5. Conclusion

This study observed that precipitation with a three month lag may be a risk factor for increased odds of malaria infection in children ages 6–59 months in Mali. The odds of testing positive for malaria by RDT for children ages 6–59 months slightly increased for every millimeter increase in total precipitation with a three month lag. Minimum, maximum, and average temperature were all statistically significantly protective with three month lag periods. Three month lag periods were statistically significant for all exposures. These results are consistent with studies conducted in other countries. Overall, these results indicate that the relationship between these environmental exposures and malaria infection in children under 5 is complex and likely differs by regional variations within the country. This exploratory study indicates the need for more regional-specific analysis in Mali to capture these complexities.

## Supporting information

S1 Fig Graphical Abstract. (DOCX)

S2 Tables Variable and Category Definitions. (DOCX)

S3 Tables Sensitivity Analysis Results. (DOCX)

S4 Tables Individual Year Results. (DOCX)

S5 Tables Effect Modification Results. (DOCX)
