# Supplementary figures and images for: Associations between precipitation, temperature, and malaria prevalence in children under 5 in Mali
Source: PLoS One. 2026 Feb 20;21(2):e0342127. doi: 10.1371/journal.pone.0342127 (PMC12923125; doi:10.1371/journal.pone.0342127)

S1 Fig. Graphical Abstract.
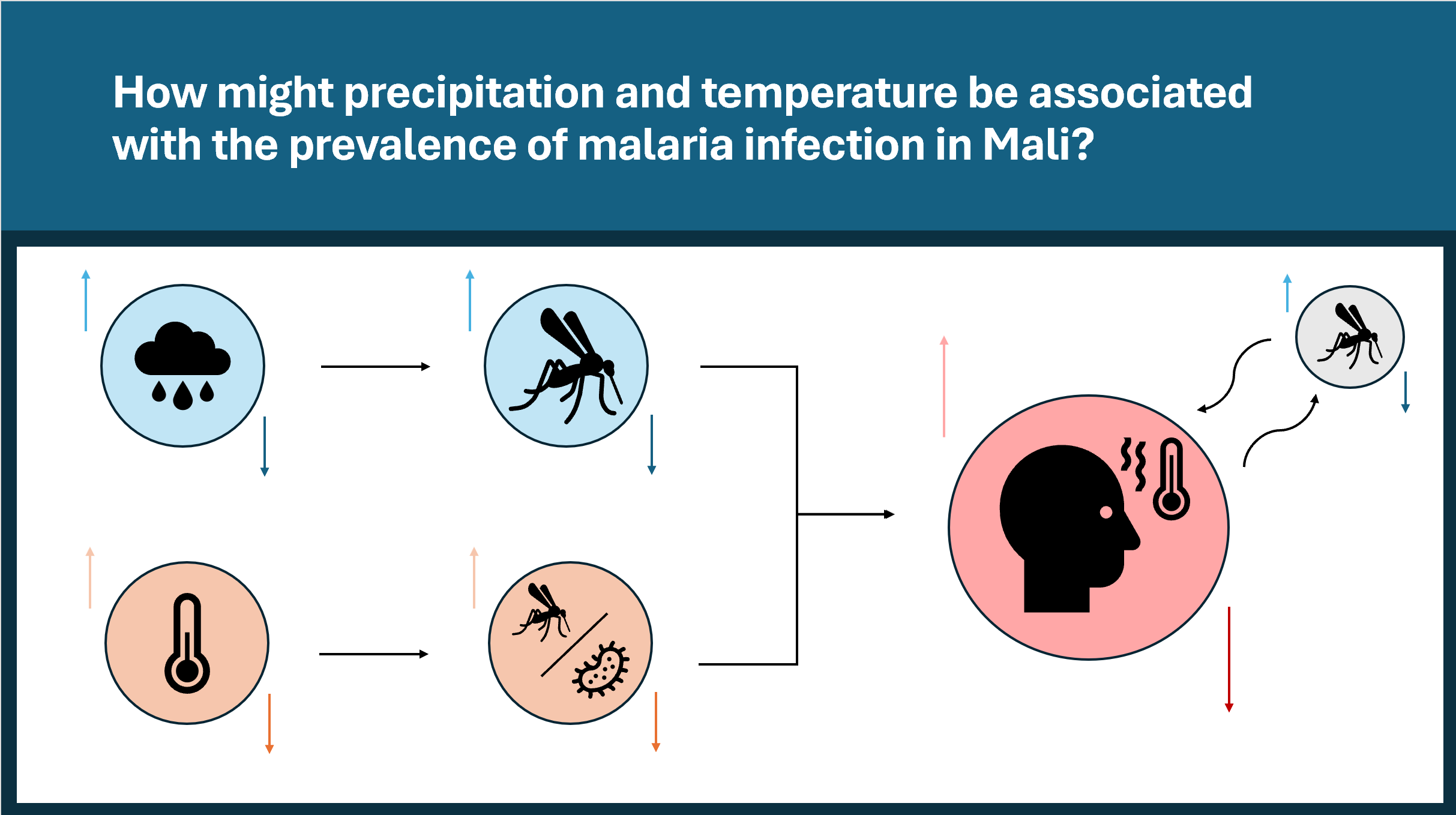

Supplement: S1 Fig — (DOCX) [file pone.0342127.s001.docx]
