# Supplementary material for: Associations between precipitation, temperature, and malaria prevalence in children under 5 in Mali
Source: PLoS One. 2026 Feb 20;21(2):e0342127. doi: 10.1371/journal.pone.0342127 (PMC12923125; doi:10.1371/journal.pone.0342127)
Supplement: S2 Tables — (DOCX) [file pone.0342127.s002.docx]

S2 Tables. Variable and Category Definitions.

Table 1. Variable category inclusions for floor, roof, and wall variables^[1,2]^.

| Natural/other floor |  |
| --- | --- |
|  | Earth/sand |
|  | Dung |
|  | Other |
| Rudimentary floor |  |
|  | Wood planks |
|  | Palm/bamboo |
| Finished floor |  |
|  | Parquet or polished wood |
|  | Vinyl or asphalt strips |
|  | Ceramic tiles |
|  | Cement |
|  | Carpet |
| Natural/other roof |  |
|  | No roof |
|  | Thatch/palm leaf |
|  | Sod |
|  | Other |
| Rudimentary roof |  |
|  | Rustic mat |
|  | Palm/bamboo |
|  | Wood planks |
|  | Cardboard |
|  | Tarpaulin/plastic |
| Finished roof |  |
|  | Metal |
|  | Wood |
|  | Zinc/calamine/cement fiber |
|  | Roof/ceramic tiles |
|  | Cement |
|  | Roofing shingles |
| Natural/other walls |  |
|  | No walls |
|  | Bamboo/cane/palm/trunks |
|  | Dirt |
|  | Other |
| Rudimentary walls |  |
|  | Bamboo with mud |
|  | Stone with mud |
|  | Uncovered adobe |
|  | Plywood |
|  | Cardboard |
|  | Reused wood |
| Finished walls |  |
|  | Cement |
|  | Stone with lime/cement |
|  | Bricks |
|  | Cement blocks |
|  | Covered adobe |
|  | Wood planks/shingles |

Table 2. Variable category inclusions for medication variables.

| Medication taken for fever – Antimalarials |  |
| --- | --- |
|  | Fansidar |
|  | Sulfadoxine pyrimethamine/fansidar |
|  | Chloroquine |
|  | Amodiaquine |
|  | Quinine |
|  | Quinine pill |
|  | Quinine injection/iv |
|  | Combination with artemisinin |
|  | Artesunate rectal |
|  | Artesunate injection/iv |
|  | Fansidar and amodiaquine (combined) |
|  | other antimalarial |
| Medication taken for fever – Antibiotics |  |
|  | Antibiotic pill/syrup |
|  | Antibiotic injection |
|  | Kunbileni |
| Medication taken for fever – Painkillers |  |
|  | Aspirin |
|  | Aspirin/paracetamol/panadol |
|  | Acetaminophen/paracetamol/panadol |
|  | Ibuprofen |
| Given medication to prevent malaria in applicable year - Antimalarials |  |
|  | Sulfadoxine pyrimethamine/fansidar and amodiaquine (both bag and package) |
|  | Fansidar and amodiaquine (both box and package) |
|  | Fansidar alone |
|  | Sulfadoxine pyrimethamine /fansidar alone |
|  | Amodiaquine alone |
|  | ACT |
|  | Chloroquine |
|  | Quinine |
|  | Other antimalarials |
| Given medication to prevent malaria in applicable year - Traditional medicine |  |
|  | Decoction/plant/root juice |
|  | Other traditional medicine |

**References**

1. The DHS Program. (2017). *Demographic and Health Surveys model household questionnaire*. https://dhsprogram.com/pubs/pdf/DHSQ7/DHS7_Household_QRE_EN_16Mar2017_DHSQ7.pdf

2. The DHS Program. (2023). *Demographic and Health Surveys model household questionnaire*. https://dhsprogram.com/pubs/pdf/DHSQ8/DHS8_Household_QRE_EN_03Feb2023_DHSQ8.pdf
