# Supplementary material for: Associations between precipitation, temperature, and malaria prevalence in children under 5 in Mali
Source: PLoS One. 2026 Feb 20;21(2):e0342127. doi: 10.1371/journal.pone.0342127 (PMC12923125; doi:10.1371/journal.pone.0342127)
Supplement: S3 Tables — (DOCX) [file pone.0342127.s003.docx]

S3 Tables. Sensitivity Analysis Results.

Table 2.1. Sensitivity analysis comparing adjusted multilevel logistic regression model associations for every lag, pooled across 2018, 2015, and 2012/13, for precipitation and temperature with malaria prevalence diagnosed by RDT in children ages 6 to 59 months, either including or excluding medication taken for fever as a covariate.

| Exposure variable | Lag | Model 1  OR (95% CI)^1^ | Model 2  OR (95% CI)^2^ |
| --- | --- | --- | --- |
| Precipitation | 0 | 1.001 (0.997, 1.005) | 1.000 (0.997, 1.004) |
|  | 1 | 0.9999 (0.995, 1.005) | 0.998 (0.993, 1.004) |
|  | 2 | 1.002 (0.997, 1.008) | 1.000 (0.995, 1.006) |
|  | 3 | **1.006 (1.002, 1.011)*** | **1.006 (1.001, 1.010)*** |
| Minimum temperature | 0 | 0.936 (0.862, 1.016) | 0.930 (0.852, 1.016) |
|  | 1 | 1.117 (0.997, 1.251) | 1.108 (0.984, 1.247) |
|  | 2 | 0.747 (0.555, 1.007) | 0.790 (0.590, 1.058) |
|  | 3 | **0.710 (0.543, 0.926)*** | **0.685 (0.513, 0.913)*** |
| Maximum temperature | 0 | 0.985 (0.891, 1.088) | 0.979 (0.888, 1.080) |
|  | 1 | 1.033 (0.938, 1.139) | 1.049 (0.953, 1.155) |
|  | 2 | **0.824 (0.682, 0.996)*** | 0.862 (0.715, 1.039) |
|  | 3 | **0.842 (0.741, 0.958)*** | **0.841 (0.733, 0.966)*** |
| Average temperature | 0 | 0 952 (0.858, 1.057) | 0.945 (0.849, 1.052) |
|  | 1 | 1.068 (0.961, 1.188) | 1.077 (0.968, 1.198) |
|  | 2 | **0.777 (0.607, 0.996)*** | 0.822 (0.644, 1.048) |
|  | 3 | **0.784 (0.654, 0.939)*** | **0.777 (0.639, 0.945)*** |

*p<0.05

1 Adjusted for Year, Sex, Urban/rural, Mothers’ education, Age, Altitude, Household electricity, Floor/Roof/Wall material, Wealth index, Hemoglobin level adjusted for altitude, Slept under a mosquito bed net the previous night

2 Adjusted for Year, Sex, Urban/rural, Mothers’ education, Age, Altitude, Household electricity, Floor/Roof/Wall material, Wealth index, Hemoglobin level adjusted for altitude, Slept under a mosquito bed net the previous night, Medication taken for fever

Table 2.2. Sensitivity analysis comparing adjusted multilevel logistic regression model associations for every lag, pooled across 2018 and 2015, for every exposure variable with malaria prevalence diagnosed by RDT in children ages 6 to 59 months, either including or excluding given medication to prevent malaria in applicable year as a covariate.

| Exposure variable | Lag | Model 1  OR (95% CI)^1^ | Model 2  OR (95% CI)^2^ |
| --- | --- | --- | --- |
| Precipitation | 0 | 1.000 (0.995, 1.005) | 1.000 (0.995, 1.005) |
|  | 1 | 0.998 (0.992, 1.003) | 0.998 (0.992, 1.003) |
|  | 2 | 0.999 (0.993, 1.005) | 0.999 (0.992, 1.005) |
|  | 3 | **1.008 (1.003, 1.014)*** | **1.008 (1.002, 1.014)*** |
| Minimum temperature | 0 | **0.840 (0.736, 0.958)*** | **0.839 (0.737, 0.956)*** |
|  | 1 | 1.045 (0.591, 1.849) | 1.049 (0.587, 1.873) |
|  | 2 | 0.800 (0.572, 1.119) | 0.800 (0.566, 1.133) |
|  | 3 | **0.708 (0.518, 0.967)*** | **0.708 (0.516, 0.972)*** |
| Maximum temperature | 0 | 0.930 (0.738, 1.171) | 0.939 (0.752, 1.172) |
|  | 1 | 1.019 (0.855, 1.214) | 1.031 (0.864, 1.231) |
|  | 2 | 0.845 (0.677, 1.056) | 0.849 (0.677, 1.067) |
|  | 3 | 0.855 (0.723, 1.010) | 0.855 (0.721, 1.014) |
| Average temperature | 0 | 0.807 (0.604, 1.077) | 0.815 (0.617, 1.077) |
|  | 1 | 1.029 (0.779, 1.359) | 1.044 (0.787, 1.385) |
|  | 2 | 0.818 (0.622, 1.076) | 0.821 (0.619, 1.089) |
|  | 3 | **0.801 (0.642, 0.999)*** | 0.801 (0.639, 1.004) |

*p<0.05

1 Adjusted for Year, Sex, Urban/rural, Mothers’ education, Age, Altitude, Household electricity, Floor/Roof/Wall material, Wealth index, Hemoglobin level adjusted for altitude, Slept under a mosquito bed net the previous night

2 Adjusted for Year, Sex, Urban/rural, Mothers’ education, Age, Altitude, Household electricity, Floor/Roof/Wall material, Wealth index, Hemoglobin level adjusted for altitude, Slept under a mosquito bed net the previous night, Given medication to prevent malaria in applicable year

Table 2.3. Sensitivity analysis comparing adjusted multilevel logistic regression model associations for every lag, pooled across 2018 and 2012/13, for every exposure variable with malaria prevalence diagnosed by RDT in children ages 6 to 59 months, either including or excluding body mass index (BMI) as a covariate.

| Exposure variable | Lag | Model 1  OR (95% CI)^1^ | Model 2  OR (95% CI)^2^ |
| --- | --- | --- | --- |
| Precipitation | 0 | 0.999 (0.996, 1.002) | 0.999 (0.996, 1.002) |
|  | 1 | 1.000 (0.989, 1.012) | 1.000 (0.989, 1.012) |
|  | 2 | 1.004 (0.997, 1.012) | 1.004 (0.996, 1.012) |
|  | 3 | **1.007 (1.000, 1.013)*** | **1.007 (1.000, 1.013)*** |
| Minimum temperature | 0 | 1.002 (0.907, 1.108) | 0.998 (0.902, 1.104) |
|  | 1 | 1.082 (0.914, 1.280) | 1.085 (0.913, 1.288) |
|  | 2 | 0.727 (0.484, 1.092) | 0.745 (0.489, 1.134) |
|  | 3 | 0.715 (0.489, 1.047) | 0.733 (0.490, 1.095) |
| Maximum temperature | 0 | 1.029 (0.946, 1.119) | 1.030 (0.946, 1.120) |
|  | 1 | 0.963 (0.796, 1.164) | 0.972 (0.799, 1.183) |
|  | 2 | 0.797 (0.620, 1.025) | 0.800 (0.618, 1.036) |
|  | 3 | **0.830 (0.696, 0.991)*** | 0.834 (0.692, 1.005) |
| Average temperature | 0 | 1.021 (0.925, 1.127) | 1.019 (0.923, 1.126) |
|  | 1 | 1.009 (0.844, 1.207) | 1.017 (0.847, 1.222) |
|  | 2 | 0.749 (0.537, 1.044) | 0.756 (0.537, 1.065) |
|  | 3 | **0.772 (0.600, 0.993)** | 0.780 (0.597, 1.018) |

*p<0.05

2 Adjusted for Year, Sex, Urban/rural, Mothers’ education, Age, Altitude, Household electricity, Floor/Roof/Wall material, Wealth index, Hemoglobin level adjusted for altitude, Slept under a mosquito bed net the previous night

2 Adjusted for Year, Sex, Urban/rural, Mothers’ education, Age, Altitude, Household electricity, Floor/Roof/Wall material, Wealth index, Hemoglobin level adjusted for altitude, Slept under a mosquito bed net the previous night, Body mass index (BMI)

Table 2.4. Sensitivity analysis comparing adjusted multilevel logistic regression model associations for every lag, pooled across 2015 and 2012/13, for every exposure variable with malaria prevalence diagnosed by RDT in children ages 6 to 59 months, either including or excluding has dwelling been sprayed in last 12 months as a covariate.

| Exposure variable | Lag | Model 1  OR (95% CI)^1^ | Model 2  OR (95% CI)^2^ |
| --- | --- | --- | --- |
| Precipitation | 0 | 1.005 (0.994, 1.017) | 1.005 (0.994, 1.017) |
|  | 1 | 1.002 (0.996, 1.008) | 1.002 (0.996, 1.008) |
|  | 2 | 1.001 (0.994, 1.009) | 1.001 (0.994, 1.009) |
|  | 3 | 1.005 (0.9997, 1.011) | 1.005 (0.9996, 1.010) |
| Minimum temperature | 0 | 0.957 (0.868, 1.054) | 0.957 (0.868, 1.054) |
|  | 1 | **1.170 (1.056, 1.297)*** | **1.170 (1.056, 1.296)*** |
|  | 2 | 0.686 (0.357, 1.318) | 0.686 (0.356, 1.320) |
|  | 3 | 0.789 (0.469, 1.325) | 0.788 (0.469, 1.325) |
| Maximum temperature | 0 | 0.990 (0.854, 1.147) | 0.990 (0.854, 1.148) |
|  | 1 | 1.082 (0.984, 1.190) | 1.082 (0.984, 1.190) |
|  | 2 | 0.840 (0.602, 1.171) | 0.841 (0.602, 1.175) |
|  | 3 | 0.875 (0.715, 1.071) | 0.876 (0.716, 1.073) |
| Average temperature | 0 | 0.967 (0.843, 1.109) | 0.967 (0.843, 1.110) |
|  | 1 | **1.126 (1.022, 1.241)** | **1.126 (1.022, 1.241)*** |
|  | 2 | 0.763 (0.465, 1.252) | 0.764 (0.465, 1.255) |
|  | 3 | 0.826 (0.603, 1.130) | 0.827 (0.604, 1.132) |

*p<0.05

1 Adjusted for Year, Sex, Urban/rural, Mothers’ education, Age, Altitude, Household electricity, Floor/Roof/Wall material, Wealth index, Hemoglobin level adjusted for altitude, Slept under a mosquito bed net the previous night

2 Adjusted for Year, Sex, Urban/rural, Mothers’ education, Age, Altitude, Household electricity, Floor/Roof/Wall material, Wealth index, Hemoglobin level adjusted for altitude, Slept under a mosquito bed net the previous night, Has dwelling been sprayed in last 12 months?

Table 2.5. Sensitivity analysis comparing adjusted multilevel logistic regression model associations for every lag, across 2021, for every exposure variable with malaria prevalence diagnosed by RDT in children ages 6 to 59 months, either including or excluding received drugs to prevent malaria this month or last as a covariate.

| Exposure variable | Lag | Model 1  OR (95% CI)^1^ | Model 2  OR (95% CI)^2^ |
| --- | --- | --- | --- |
| Precipitation | 0 | **0.993 (0.987, 0.999)*** | **0.993 (0.987, 0.9998)*** |
|  | 1 | 1.003 (0.998, 1.008) | 1.003 (0.998, 1.007) |
|  | 2 | 0.996 (0.990, 1.002) | 0.996 (0.990, 1.002) |
|  | 3 | 0.999 (0.989, 1.009) | 0.999 (0.990, 1.009) |
| Minimum temperature | 0 | **1.427 (1.001, 2.033)*** | **1.445 (1.010, 2.068)*** |
|  | 1 | 1.127 (0.817, 1.554) | 1.111 (0.810, 1.523) |
|  | 2 | 1.336 (0.801, 2.229) | 1.307 (0.810, 2.110) |
|  | 3 | 1.090 (0.676, 1.757) | 1.067 (0.683, 1.665) |
| Maximum temperature | 0 | 1.006 (0.853, 1.188) | 0.985 (0.834, 1.164) |
|  | 1 | 0.869 (0.687, 1.101) | 0.868 (0.695, 1.084) |
|  | 2 | 1.087 (0.807, 1.464) | 1.069 (0.808, 1.415) |
|  | 3 | 1.038 (0.832, 1.295) | 1.023 (0.832, 1.259) |
| Average temperature | 0 | 1.135 (0.872, 1.476) | 1.115 (0.855, 1.455) |
|  | 1 | 0.877 (0.644, 1.193) | 0.873 (0.654, 1.167) |
|  | 2 | 1.170 (0.784, 1.745) | 1.148 (0.789, 1.670) |
|  | 3 | 1.056 (0.771, 1.447) | 1.037 (0.773, 1.391) |

*p<0.05

1 Adjusted for Sex, Urban/rural, Mothers’ education, Age, Altitude, Household electricity, Floor/Roof/Wall material, Wealth index, Hemoglobin level adjusted for altitude, Slept under a mosquito bed net the previous night

2 Adjusted for Sex, Urban/rural, Mothers’ education, Age, Altitude, Household electricity, Floor/Roof/Wall material, Wealth index, Hemoglobin level adjusted for altitude, Slept under a mosquito bed net the previous night, Received drugs to prevent malaria this month or last
