# Supplementary material for: Associations between precipitation, temperature, and malaria prevalence in children under 5 in Mali
Source: PLoS One. 2026 Feb 20;21(2):e0342127. doi: 10.1371/journal.pone.0342127 (PMC12923125; doi:10.1371/journal.pone.0342127)
Supplement: S4 Tables — (DOCX) [file pone.0342127.s004.docx]

S4 Tables. Individual Year Results.

Table 3.1. Adjusted multilevel logistic regression model associations for 0-3 month lags for 2021 for precipitation and temperature with malaria prevalence diagnosed by RDT in children ages 6 to 59 months.

| Exposure variable | Lag | Model 1  OR (95% CI) | Model 2  OR (95% CI)^1^ | Model 3  OR (95% CI)^2^ | Model 4  OR (95% CI)^3^ | Model 5  OR (95% CI)^4^ |
| --- | --- | --- | --- | --- | --- | --- |
| Precipitation | 0 | **0.992 (0.988, 0.997)*** | **0.992 (0.986, 0.998)*** | **0.993 (0.987, 0.999)*** | **0.993 (0.987, 0.999)*** | **0.993 (0.987, 0.9998)*** |
|  | 1 | 1.001 (0.997, 1.005) | 1.002 (0.997, 1.008) | 1.002 (0.997, 1.007) | 1.003 (0.998, 1.008) | 1.003 (0.998, 1.007) |
|  | 2 | 0.998 (0.994, 1.002) | 0.997 (0.990, 1.003) | 0.997 (0.990, 1.004) | 0.996 (0.990, 1.002) | 0.996 (0.990, 1.002) |
|  | 3 | 1.001 (0.992, 1.009) | 1.001 (0.990, 1.012) | 1.001 (0.992, 1.011) | 0.999 (0.989, 1.009) | 0.999 (0.990, 1.009) |
| Minimum temperature | 0 | **1.437 (1.157, 1.785)*** | **1.503 (1.076, 2.099)*** | **1.426 (1.003, 2.029)*** | **1.427 (1.001, 2.034)*** | **1.445 (1.010, 2.068)*** |
|  | 1 | 1.219 (0.977, 1.522) | 1.260 (0.892, 1.780) | 1.209 (0.853, 1.714) | 1.126 (0.818, 1.550) | 1.111 (0.810, 1.523) |
|  | 2 | 1.105 (0.789, 1.549) | 1.290 (0.749, 2.222) | 1.243 (0.738, 2.093) | 1.335 (0.802, 2.221) | 1.307 (0.810, 2.110) |
|  | 3 | 1.003 (0.758, 1.327) | 1.063 (0.644, 1.756) | 1.022 (0.640, 1.631) | 1.089 (0.677, 1.753) | 1.067 (0.683, 1.665) |
| Maximum temperature | 0 | 0.985 (0.877, 1.107) | 1.032 (0.879, 1.211) | 1.010 (0.854, 1.194) | 1.007 (0.853, 1.188) | 0.985 (0.834, 1.164) |
|  | 1 | 0.957 (0.797, 1.149) | 0.922 (0.700, 1.215) | 0.921 (0.716, 1.183) | 0.869 (0.688, 1.099) | 0.868 (0.695, 1.084) |
|  | 2 | 0.978 (0.799, 1.198) | 1.043 (0.750, 1.452) | 1.023 (0.752, 1.391) | 1.086 (0.807, 1.461) | 1.069 (0.808, 1.415) |
|  | 3 | 0.977 (0.833, 1.146) | 0.9996 (0.788, 1.267) | 0.986 (0.792, 1.228) | 1.038 (0.833, 1.294) | 1.023 (0.832, 1.259) |
| Average temperature | 0 | 1.118 (0.948, 1.318) | 1.185 (0.922, 1.524) | 1.139 (0.874, 1.485) | 1.135 (0.872, 1.477) | 1.115 (0.855, 1.455) |
|  | 1 | 1.002 (0.803, 1.250) | 0.956 (0.673, 1.359) | 0.947 (0.684, 1.310) | 0.877 (0.646, 1.190) | 0.873 (0.654, 1.167) |
|  | 2 | 1.018 (0.779, 1.329) | 1.123 (0.724, 1.744) | 1.093 (0.723, 1.652) | 1.169 (0.786, 1.740) | 1.148 (0.789, 1.670) |
|  | 3 | 0.980 (0.794, 1.210) | 1.013 (0.725, 1.416) | 0.991 (0.727, 1.352) | 1.056 (0.772, 1.444) | 1.037 (0.773, 1.391) |

*p<0.05

1 Adjusted for Sex, Urban/rural, Mothers’ education, Age, Altitude

2 Adjusted for Sex, Urban/rural, Mothers’ education, Age, Altitude, Household electricity, Floor/Roof/Wall material, Wealth index

3 Adjusted for Sex, Urban/rural, Mothers’ education, Age, Altitude, Household electricity, Floor/Roof/Wall material, Wealth index, Hemoglobin level adjusted for altitude

4 Adjusted for Sex, Urban/rural, Mothers’ education, Age, Altitude, Household electricity, Floor/Roof/Wall material, Wealth index, Hemoglobin level adjusted for altitude, Slept under a mosquito bed net the previous night, Received drugs to prevent malaria this month or last

Table 3.2. Adjusted multilevel logistic regression model associations for every lag of 2018 for every exposure variable with malaria prevalence diagnosed by RDT in children ages 6 to 59 months.

| Exposure variable | Lag | Model 1  OR (95% CI) | Model 2  OR (95% CI)^1^ | Model 3  OR (95% CI)^2^ | Model 4  OR (95% CI)^3^ | Model 5  OR (95% CI)^4^ |
| --- | --- | --- | --- | --- | --- | --- |
| Precipitation | 0 | 0.999 (0.996, 1.003) | **0.997 (0.993, 0.9997)*** | 0.997 (0.994, 1.000) | 0.997 (0.994, 1.000) | 0.997 (0.994, 1.001) |
|  | 1 | 0.992 (0.979, 1.006) | 0.991 (0.974, 1.008) | 0.992 (0.976, 1.008) | 0.991 (0.974, 1.009) | 0.988 (0.967, 1.009) |
|  | 2 | 1.001 (0.991, 1.011) | 1.001 (0.989, 1.013) | 1.001 (0.990, 1.012) | 0.999 (0.987, 1.011) | 0.999 (0.986, 1.013) |
|  | 3 | 1.008 (0.999, 1.017) | 1.009 (0.998, 1.019) | **1.010 (1.000, 1.019)*** | 1.009 (0.997, 1.020) | 1.011 (0.997, 1.025) |
| Minimum temperature | 0 | 0.892 (0.735, 1.081) | **1.427 (1.014, 2.009)*** | 1.317 (0.939, 1.846) | 1.355 (0.952, 1.928) | 1.339 (0.933, 1.919) |
|  | 1 | 0.990 (0.465, 2.108) | 1.065 (0.341, 3.325) | 0.971 (0.324, 2.909) | 0.979 (0.294, 3.265) | 1.146 (0.347, 3.792) |
|  | 2 | 0.838 (0.580, 1.211) | 0.858 (0.534, 1.380) | 0.832 (0.531, 1.303) | 0.953 (0.576, 1.577) | 1.001 (0.596, 1.680) |
|  | 3 | 0.714 (0.485, 1.053) | 0.703 (0.413, 1.198) | 0.682 (0.414, 1.124) | 0.737 (0.402, 1.353) | 0.687 (0.330, 1.429) |
| Maximum temperature | 0 | 0.905 (0.804 ,1.019) | 1.154 (0.987, 1.349) | 1.114 (0.952, 1.304) | 1.130 (0.961, 1.327) | 1.110 (0.943, 1.308) |
|  | 1 | 0.995 (0.667, 1.483) | 1.005 (0.599, 1.688) | 0.976 (0.581, 1.641) | 1.007 (0.561, 1.806) | 1.058 (0.599, 1.870) |
|  | 2 | 0.839 (0.644, 1.093) | 0.843 (0.614, 1.157) | 0.823 (0.609, 1.112) | 0.889 (0.627, 1.260) | 0.896 (0.608, 1.322) |
|  | 3 | 0.849 (0.676, 1.066) | 0.851 (0.642, 1.130) | 0.831 (0.634, 1.089) | 0.878 (0.639, 1.206) | 0.862 (0.594, 1.251) |
| Average temperature | 0 | 0.870 (0.740, 1.023) | **1.328 (1.031, 1.710)*** | 1.242 (0.959, 1.609) | 1.274 (0.973, 1.669) | 1.243 (0.946, 1.635) |
|  | 1 | 0.993 (0.587, 1.680) | 1.021 (0.496, 2.100) | 0.970 (0.473, 1.988) | 1.002 (0.449, 2.232) | 1.090 (0.503, 2.366) |
|  | 2 | 0.825 (0.602, 1.130) | 0.833 (0.565, 1.227) | 0.810 (0.561, 1.169) | 0.895 (0.588, 1.363) | 0.914 (0.580, 1.442) |
|  | 3 | 0.798 (0.596, 1.068) | 0.797 (0.547, 1.161) | 0.774 (0.542, 1.107) | 0.827 (0.540, 1.267) | 0.800 (0.483, 1.327) |

*p<0.05

1 Adjusted for Sex, Urban/rural, Mothers’ education, Age, Altitude

2 Adjusted for Sex, Urban/rural, Mothers’ education, Age, Altitude, Household electricity, Floor/Roof/Wall material, Wealth index

3 Adjusted for Sex, Urban/rural, Mothers’ education, Age, Altitude, Household electricity, Floor/Roof/Wall material, Wealth index, Hemoglobin level adjusted for altitude, BMI

4 Adjusted for Sex, Urban/rural, Mothers’ education, Age, Altitude, Household electricity, Floor/Roof/Wall material, Wealth index, Hemoglobin level adjusted for altitude, BMI, Slept under a mosquito bed net the previous night, Medication taken for fever, Given medication to prevent malaria in applicable year

Table 3.3. Adjusted multilevel logistic regression model associations for every lag of 2015 for every exposure variable with malaria prevalence diagnosed by RDT in children ages 6 to 59 months.

| Exposure variable | Lag | Model 1  OR (95% CI) | Model 2  OR (95% CI)^1^ | Model 3  OR (95% CI)^2^ | Model 4  OR (95% CI)^3^ | Model 5  OR (95% CI)^4^ |
| --- | --- | --- | --- | --- | --- | --- |
| Precipitation | 0 | 1.003 (0.979, 1.027) | 1.009 (0.993, 1.025) | 1.009 (0.993, 1.026) | 1.007 (0.993, 1.022) | 1.005 (0.992, 1.019) |
|  | 1 | 0.9996 (0.993, 1.006) | 1.001 (0.995, 1.006) | 1.000 (0.994, 1.006) | 0.9995 (0.995, 1.004) | 0.999 (0.994, 1.005) |
|  | 2 | **1.002 (1.000, 1.004)*^5^** | 0.994 (0.987, 1.000) | 0.993 (0.985, 1.001) | 0.994 (0.987, 1.001) | 0.993 (0.985, 1.000) |
|  | 3 | 1.008 (0.997, 1.019) | 1.006 (0.996, 1.017) | 1.008 (0.997, 1.019) | 1.008 (0.999, 1.016) | 1.008 (0.999, 1.017) |
| Minimum temperature | 0 | 0.889 (0.761, 1.040) | **0.805 (0.678, 0.955)*** | **0.820 (0.690, 0.976)*** | **0.813 (0.688, 0.960)*** | **0.810 (0.687, 0.955)*** |
|  | 1 | 1.185 (0.671, 2.093) | 1.005 (0.494, 2.044) | 1.058 (0.494, 2.269) | 1.124 (0.643, 1.966) | 1.240 (0.684, 2.248) |
|  | 2 | **0.744 (0.603, 0.918)*^5^** | 1.162 (0.239, 5.643) | 1.091 (0.192, 6.205) | 0.875 (0.152, 5.040) | 0.927 (0.157, 5.488) |
|  | 3 | 0.825 (0.414, 1.642) | 0.871 (0.451, 1.683) | 0.799 (0.390, 1.640) | 0.730 (0.355, 1.500) | 0.721 (0.340, 1.530) |
| Maximum temperature | 0 | 0.883 (0.430, 1.812) | 0.636 (0.345, 1.172) | 0.627 (0.334, 1.177) | 0.673 (0.389, 1.163) | 0.731 (0.450, 1.188) |
|  | 1 | 1.036 (0.779, 1.376) | 0.972 (0.758, 1.247) | 1.008 (0.767, 1.324) | 1.076 (0.902, 1.282) | 1.116 (0.941, 1.324) |
|  | 2 | **0.776 (0.666, 0.905)*^5^** | 0.991 (0.515, 1.908) | 0.985 (0.477, 2.030) | 0.996 (0.442, 2.244) | 1.043 (0.450, 2.419) |
|  | 3 | 0.930 (0.668, 1.295) | 0.972 (0.720, 1.311) | 0.938 (0.675, 1.303) | 0.898 (0.633, 1.274) | 0.892 (0.617, 1.291) |
| Average temperature | 0 | 0.732 (0.282, 1.900) | **0.475 (0.236, 0.953)*** | **0.481 (0.232, 0.999)*** | **0.508 (0.276, 0.935)*** | **0.547 (0.322, 0.929)*** |
|  | 1 | 1.069 (0.709, 1.611) | 0.965 (0.651, 1.432) | 1.016 (0.665, 1.553) | 1.112 (0.859, 1.439) | 1.178 (0.915, 1.518) |
|  | 2 | **0.747 (0.621, 0.898)*^5^** | 1.018 (0.384, 2.696) | 0.999 (0.339, 2.945) | 0.971 (0.294, 3.203) | 1.031 (0.302, 3.526) |
|  | 3 | 0.898 (0.570, 1.414) | 0.947 (0.625, 1.434) | 0.900 (0.571, 1.419) | 0.847 (0.525, 1.365) | 0.840 (0.509, 1.388) |

*p<0.05

1 Adjusted for Sex, Urban/rural, Mothers’ education, Age, Altitude

2 Adjusted for Sex, Urban/rural, Mothers’ education, Age, Altitude, Household electricity, Floor/Roof/Wall material, Wealth index

3 Adjusted for Sex, Urban/rural, Mothers’ education, Age, Altitude, Household electricity, Floor/Roof/Wall material, Wealth index, Hemoglobin level adjusted for altitude

4 Adjusted for Sex, Urban/rural, Mothers’ education, Age, Altitude, Household electricity, Floor/Roof/Wall material, Wealth index, Hemoglobin level adjusted for altitude, Slept under a mosquito bed net the previous night, Medication taken for fever, Given medication to prevent malaria in applicable year, Has dwelling been sprayed in last 12 months?

5 Lag 2 was not run as a multilevel model for the univariate analysis due to initial values being unfeasible

Table 3.4. Adjusted multilevel logistic regression model associations for every lag of 2012/13 for every exposure variable with malaria prevalence diagnosed by RDT in children ages 6 to 59 months.

| Exposure variable | Lag | Model 1  OR (95% CI) | Model 2  OR (95% CI)^1^ | Model 3  OR (95% CI)^2^ | Model 4  OR (95% CI)^3^ | Model 5  OR (95% CI)^4^ |
| --- | --- | --- | --- | --- | --- | --- |
| Precipitation | 0 | 1.014 (0.911, 1.129) | 1.053 (0.929, 1.193) | 1.059 (0.940, 1.193) | 1.051 (0.938, 1.177) | 1.048 (0.925, 1.188) |
|  | 1 | 1.008 (0.993, 1.024) | 1.008 (0.992, 1.024) | 1.008 (0.991, 1.025) | 1.009 (0.989, 1.028) | 1.000 (0.981, 1.020) |
|  | 2 | 1.005 (0.995, 1.014) | 1.005 (0.995, 1.015) | 1.005 (0.995, 1.016) | 1.006 (0.994, 1.018) | 1.001 (0.989, 1.013) |
|  | 3 | 1.004 (0.997, 1.012) | 1.004 (0.997, 1.012) | 1.005 (0.996, 1.013) | 1.006 (0.997, 1.015) | 1.003 (0.996, 1.011) |
| Minimum temperature | 0 | 0.940 (0.838, 1.053) | 1.045 (0.916, 1.193) | 1.053 (0.926, 1.199) | 1.027 (0.902, 1.170) | 1.034 (0.893, 1.198) |
|  | 1 | 1.025 (0.804, 1.306) | 1.078 (0.847, 1.373) | 1.097 (0.883, 1.361) | 1.129 (0.950, 1.342) | 1.100 (0.910, 1.329) |
|  | 2 | 0.557 (0.222, 1.399) | 0.495 (0.162, 1.518) | 0.486 (0.145, 1.626) | 0.452 (0.123, 1.659) | 0.666 (0.219, 2.028) |
|  | 3 | 1.650 (0.664, 4.102) | 1.697 (0.482, 5.974) | 1.573 (0.430, 5.762) | 1.552 (0.379, 6.360) | 1.084 (0.376, 3.128) |
| Maximum temperature | 0 | 0.924 (0.825, 1.035) | 1.124 (0.984, 1.284) | 1.124 (0.987, 1.280) | 1.081 (0.949, 1.231) | 1.074 (0.928, 1.244) |
|  | 1 | 0.845 (0.616, 1.160) | 0.931 (0.699, 1.242) | 0.949 (0.718, 1.253) | 0.952 (0.718, 1.263) | 0.9999 (0.788, 1.268) |
|  | 2 | 0.802 (0.521, 1.234) | 0.773 (0.495, 1.206) | 0.762 (0.474, 1.226) | 0.740 (0.440, 1.245) | 0.890 (0.541, 1.463) |
|  | 3 | 0.966 (0.740, 1.260) | 0.897 (0.676, 1.189) | 0.881 (0.669, 1.162) | 0.827 (0.654, 1.045) | 0.869 (0.703, 1.074) |
| Average temperature | 0 | 0.927 (0.824, 1.043) | 1.089 (0.948, 1.251) | 1.094 (0.956, 1.253) | 1.057 (0.923, 1.211) | 1.057 (0.907, 1.233) |
|  | 1 | 0.925 (0.722, 1.185) | 0.9996 (0.781, 1.280) | 1.018 (0.810, 1.280) | 1.037 (0.852, 1.260) | 1.048 (0.866, 1.268) |
|  | 2 | 0.705 (0.373, 1.330) | 0.659 (0.329, 1.319) | 0.645 (0.307, 1.356) | 0.617 (0.275, 1.383) | 0.812 (0.375, 1.758) |
|  | 3 | 1.053 (0.744, 1.492) | 0.913 (0.582, 1.431) | 0.879 (0.575, 1.344) | 0.788 (0.575, 1.079) | 0.809 (0.583, 1.124) |

*p<0.05

1 Adjusted for Sex, Urban/rural, Mothers’ education, Age, Altitude

2 Adjusted for Sex, Urban/rural, Mothers’ education, Age, Altitude, Household electricity, Floor/Roof/Wall material, Wealth index

3 Adjusted for Sex, Urban/rural, Mothers’ education, Age, Altitude, Household electricity, Floor/Roof/Wall material, Wealth index, Hemoglobin level adjusted for altitude, BMI

4 Adjusted for Sex, Urban/rural, Mothers’ education, Age, Altitude, Household electricity, Floor/Roof/Wall material, Wealth index, Hemoglobin level adjusted for altitude, BMI, Slept under a mosquito bed net the previous night, Medication taken for fever, Has dwelling been sprayed in last 12 months?
