# Supplementary material for: Associations between precipitation, temperature, and malaria prevalence in children under 5 in Mali
Source: PLoS One. 2026 Feb 20;21(2):e0342127. doi: 10.1371/journal.pone.0342127 (PMC12923125; doi:10.1371/journal.pone.0342127)
Supplement: S5 Tables — (DOCX) [file pone.0342127.s005.docx]

S5 Tables. Effect Modification Results.

Table 4.1. Adjusted multilevel logistic regression model associations for every lag, pooled across 2021, 2018, 2015, and 2012/13, for every exposure variable with malaria prevalence diagnosed by RDT in children ages 6 to 59 months including adjustment for sex and age as potential effect modification.

| Exposure variable | Lag | Model 1  OR (95% CI)^1^ | Model 2  OR (95% CI)^2^ |
| --- | --- | --- | --- |
| Precipitation | 0 | Male: (ref)  Female: 0.9999 (0.999, 1.001) | 6-11 month: (ref)  12-23 months: **1.003 (1.001, 1.005)***  24-35 months: 1.002 (0.999, 1.005)  36-47 months: **1.003 (1.001, 1.005)***  48-59 months: 1.002 (0.9996, 1.005) |
|  | 1 | Male: (ref)  Female: 0.9999 (0.999, 1.001) | 6-11 months: (ref)  12-23 months: **1.002 (1.000, 1.004)***  24-35 months: 1.001 (0.9997, 1.003)  36-47 months: **1.003 (1.001, 1.004)***  48-59 months: 1.001 (0.999, 1.003) |
|  | 2 | Male: (ref)  Female: 0.9997 (0.999, 1.001) | 6-11 months: (ref)  12-23 months: 1.001 (0.9997, 1.003)  24-35 months: 1.001 (0.9995, 1.003)  36-47 months: **1.002 (1.000, 1.003)***  48-59 months: 1.000 (0.998, 1.002) |
|  | 3 | Male: (ref)  Female: 1.000 (0.999, 1.002) | 6-11 months: (ref)  12-23 months: 1.000 (0.998, 1.002)  24-35 months: 1.000 (0.998, 1.003)  36-47 months: 1.001 (0.999, 1.003)  48-59 months: 1.001 (0.999, 1.004) |
| Minimum temperature | 0 | Male: (ref)  Female: 0.998 (0.960, 1.038) | 6-11 months: (ref)  12-23 months: 1.015 (0.941, 1.094)  24-35 months: 1.061 (0.983, 1.146)  36-47 months: 1.068 (0.992, 1.150)  48-59 months: 0.980 (0.909, 1.057) |
|  | 1 | Male: (ref)  Female: 1.038 (0.984, 1.095) | 6-11 months: (ref)  12-23 months: 0.962 (0.858, 1.079)  24-35 months: 1.057 (0.945, 1.183)  36-47 months: 1.072 (0.961, 1.196)  48-59 months: 0.986 (0.883, 1.102) |
|  | 2 | Male: (ref)  Female: 1.036 (0.944, 1.136) | 6-11 months: (ref)  12-23 months: 0.929 (0.788, 1.094)  24-35 months: 0.980 (0.833, 1.153)  36-47 months: 1.007 (0.861, 1.178)  48-59 months: 0.961 (0.820, 1.127) |
|  | 3 | Male: (ref)  Female: 0.990 (0.924, 1.061) | 6-11 months: (ref)  12-23 months: 0.958 (0.845, 1.085)  24-35 months: 1.013 (0.897, 1.145)  36-47 months: 1.035 (0.922, 1.163)  48-59 months: 0.944 (0.835, 1.067) |
| Maximum temperature | 0 | Male: (ref)  Female: 1.003 (0.968, 1.040) | 6-11 months: (ref)  12-23 months: **0.927 (0.859, 0.9998)***  24-35 months: 1.007 (0.930, 1.090)  36-47 months: 0.981 (0.909, 1.059)  48-59 months: 0.935 (0.868, 1.007) |
|  | 1 | Male: (ref)  Female: 1.026 (0.985, 1.068) | 6-11 months: (ref)  12-23 months: **0.911 (0.837, 0.991)***  24-35 months: 0.985 (0.903, 1.074)  36-47 months: 0.952 (0.873, 1.037)  48-59 months: 0.952 (0.878, 1.033) |
|  | 2 | Male: (ref)  Female: 1.019 (0.967, 1.073) | 6-11 months: (ref)  12-23 months: 0.957 (0.877, 1.046)  24-35 months: 0.948 (0.865, 1.039)  36-47 months: 0.960 (0.879, 1.048)  48-59 months: 0.976 (0.896, 1.063) |
|  | 3 | Male: (ref)  Female: 0.995 (0.955, 1.037) | 6-11 months: (ref)  12-23 months: 0.992 (0.924, 1.064)  24-35 months: 0.994 (0.926, 1.066)  36-47 months: 1.014 (0.950, 1.082)  48-59 months: 0.965 (0.899, 1.035) |
| Average temperature | 0 | Male: (ref)  Female: 1.001 (0.960, 1.044) | 6-11 months: (ref)  12-23 months: 0.960 (0.880, 1.047)  24-35 months: 1.040 (0.952, 1.137)  36-47 months: 1.026 (0.941, 1.119)  48-59 months: 0.946 (0.868, 1.031) |
|  | 1 | Male: (ref)  Female: 1.035 (0.986, 1.088) | 6-11 months: (ref)  12-23 months: 0.918 (0.825, 1.023)  24-35 months: 1.014 (0.910, 1.129)  36-47 months: 0.996 (0.894, 1.108)  48-59 months: 0.960 (0.866, 1.064) |
|  | 2 | Male: (ref)  Female: 1.028 (0.957, 1.103) | 6-11 months: (ref)  12-23 months: 0.939 (0.833, 1.059)  24-35 months: 0.946 (0.836, 1.071)  36-47 months: 0.964 (0.857, 1.085)  48-59 months: 0.964 (0.858, 1.084) |
|  | 3 | Male: (ref)  Female: 0.993 (0.941, 1.047) | 6-11 months: (ref)  12-23 months: 0.981 (0.893, 1.076)  24-35 months: 0.999 (0.911, 1.095)  36-47 months: 1.021 (0.938, 1.112)  48-59 months: 0.955 (0.871, 1.047) |

*p<0.05

1 Adjusted for Year, Sex, Urban/rural, Mothers’ education, Age, Altitude, Household electricity, Floor/Roof/Wall material, Wealth index, Hemoglobin level adjusted for altitude, Slept under a mosquito bed net the previous night, Sex*Precipitation. Results presented for Sex*Exposure

2 Adjusted for Year, Sex, Urban/rural, Mothers’ education, Age, Altitude, Household electricity, Floor/Roof/Wall material, Wealth index, Hemoglobin level adjusted for altitude, Slept under a mosquito bed net the previous night, Age*Precipitation. Results presented for Age*Exposure

Table 4.2. Adjusted multilevel logistic regression model associations for every lag of 2021 for every exposure variable with malaria prevalence diagnosed by RDT in children ages 6 to 59 months including adjustment for sex and age as potential effect modification.

| Exposure variable | Lag | Model 1  OR (95% CI)^1^ | Model 2  OR (95% CI)^2^ |
| --- | --- | --- | --- |
| Precipitation | 0 | Male: (ref)  Female: 1.000 (0.997, 1.003) | 6-11 months: (ref)  12-23 months: **1.007 (1.001, 1.013)***  24-35 months: 1.003 (0.997, 1.009)  36-47 months: 1.003 (0.997, 1.009)  48-59 months: 1.004 (0.998, 1.010) |
|  | 1 | Male: (ref)  Female: 1.001 (0.999, 1.003) | 6-11 months: (ref)  12-23 months: **1.006 (1.001, 1.010)***  24-35 months: 1.004 (0.999, 1.008)  36-47 months: 1.004 (0.99998, 1.009)  48-59 months: **1.004 (1.000, 1.009)*** |
|  | 2 | Male: (ref)  Female: 0.9998 (0.998, 1.002) | 6-11 months: (ref)  12-23 months: 1.002 (0.999, 1.006)  24-35 months: 1.002 (0.998, 1.006)  36-47 months: 0.9996 (0.996, 1.004)  48-59 months: 1.001 (0.995, 1.007) |
|  | 3 | Male: (ref)  Female: 0.999 (0.995, 1.002) | 6-11 months: (ref)  12-23 months: 0.998 (0.992, 1.004)  24-35 months: 0.999 (0.994, 1.005)  36-47 months: **0.994 (0.989, 0.9998)***  48-59 months: 0.999 (0.994, 1.004) |
| Minimum temperature | 0 | Male: (ref)  Female: 1.047 (0.878, 1.249) | 6-11 months: (ref)  12-23 months: 0.861 (0.659, 1.124)  24-35 months: 0.922 (0.708, 1.199)  36-47 months: 1.043 (0.805, 1.350)  48-59 months: 0.892 (0.696, 1.142) |
|  | 1 | Male: (ref)  Female: 0.999 (0.841, 1.187) | 6-11 months: (ref)  12-23 months: 0.699 (0.484, 1.010)  24-35 months: 0.780 (0.536, 1.136)  36-47 months: 0.839 (0.576, 1.222)  48-59 months: 0.705 (0.493, 1.008) |
|  | 2 | Male: (ref)  Female: 1.055 (0.879, 1.266) | 6-11 months: (ref)  12-23 months: 1.046 (0.772, 1.417)  24-35 months: 0.986 (0.746, 1.304)  36-47 months: 1.215 (0.897, 1.646)  48-59 months: 0.979 (0.745, 1.285) |
|  | 3 | Male: (ref)  Female: 1.017 (0.886, 1.167) | 6-11 months: (ref)  12-23 months: 0.925 (0.727, 1.176)  24-35 months: 0.931 (0.744, 1.165)  36-47 months: 1.060 (0.838, 1.341)  48-59 months: 0.905 (0.725, 1.131) |
| Maximum temperature | 0 | Male: (ref)  Female: 0.983 (0.908, 1.065) | 6-11 months: (ref)  12-23 months: **0.810 (0.665, 0.987)***  24-35 months: 0.868 (0.707, 1.066)  36-47 months: 0.857 (0.698, 1.053)  48-59 months: **0.830 (0.691, 0.998)*** |
|  | 1 | Male: (ref)  Female: 0.969 (0.897, 1.047) | 6-11 months: (ref)  12-23 months: **0.789 (0.643, 0.969)***  24-35 months: 0.846 (0.681, 1.051)  36-47 months: 0.828 (0.667, 1.027)  48-59 months: **0.790 (0.648, 0.963)*** |
|  | 2 | Male: (ref)  Female: 1.024 (0.920, 1.139) | 6-11 months: (ref)  12-23 months: 1.034 (0.849, 1.260)  24-35 months: 0.992 (0.826, 1.191)  36-47 months: 1.128 (0.929, 1.369)  48-59 months: 0.972 (0.815, 1.159) |
|  | 3 | Male: (ref)  Female: 1.019 (0.943, 1.100) | 6-11 months: (ref)  12-23 months: 1.036 (0.905, 1.185)  24-35 months: 1.005 (0.888, 1.137)  36-47 months: 1.100 (0.966, 1.253)  48-59 months: 0.984 (0.872, 1.112) |
| Average temperature | 0 | Male: (ref)  Female: 0.999 (0.887, 1.124) | 6-11 months: (ref)  12-23 months: 0.783 (0.609, 1.005)  24-35 months: 0.853 (0.660, 1.103)  36-47 months: 0.877 (0.678, 1.134)  48-59 months: 0.810 (0.642, 1.023) |
|  | 1 | Male: (ref)  Female: 0.971 (0.870, 1.084) | 6-11 months: (ref)  12-23 months: **0.728 (0.553, 0.959)***  24-35 months: 0.799 (0.598, 1.066)  36-47 months: 0.798 (0.599, 1.063)  48-59 months: **0.731 (0.559, 0.955)*** |
|  | 2 | Male: (ref)  Female: 1.035 (0.902, 1.187) | 6-11 months: (ref)  12-23 months: 1.041 (0.817, 1.327)  24-35 months: 0.990 (0.791, 1.238)  36-47 months: 1.165 (0.917, 1.480)  48-59 months: 0.972 (0.783, 1.207) |
|  | 3 | Male: (ref)  Female: 1.020 (0.922, 1.129) | 6-11 months: (ref)  12-23 months: 1.011 (0.845, 1.209)  24-35 months: 0.986 (0.837, 1.162)  36-47 months: 1.103 (0.928, 1.312)  48-59 months: 0.962 (0.817, 1.131) |

*p<0.05

1 Adjusted for Sex, Urban/rural, Mothers’ education, Age, Altitude, Household electricity, Floor/Roof/Wall material, Wealth index, Hemoglobin level adjusted for altitude, Slept under a mosquito bed net the previous night, Received drugs to prevent malaria this month or last, Sex*Exposure. Results presented for Sex*Exposure

2 Adjusted for Sex, Urban/rural, Mothers’ education, Age, Altitude, Household electricity, Floor/Roof/Wall material, Wealth index, Hemoglobin level adjusted for altitude, Slept under a mosquito bed net the previous night, Received drugs to prevent malaria this month or last, Age*Exposure. Results presented for Age*Exposure

Table 4.3. Adjusted multilevel logistic regression model associations for every lag of 2018 for every exposure variable with malaria prevalence diagnosed by RDT in children ages 6 to 59 months including adjustment for sex and age as potential effect modification.

| Exposure variable | Lag | Model 1  OR (95% CI)^1^ | Model 2  OR (95% CI)^2^ |
| --- | --- | --- | --- |
| Precipitation | 0 | Male: (ref)  Female: 1.000 (0.997, 1.003) | 6-11 months: (ref)  12-23 months: 1.000 (0.994, 1.006)  24-35 months: 1.000 (0.994, 1.007)  36-47 months: 0.999 (0.994, 1.004)  48-59 months: 1.002 (0.996, 1.008) |
|  | 1^3^ | Male: (ref)  Female: 1.002 (0.999, 1.005) | 6-11 months: (ref)  12-23 months: 1.002 (0.996, 1.007)  24-35 months: 0.997 (0.990, 1.004)  36-47 months: 1.002 (0.996, 1.007)  48-59 months: 0.999 (0.992, 1.005) |
|  | 2 | Male: (ref)  Female: 0.9997 (0.996, 1.004) | 6-11 months: (ref)  12-23 months: 0.999 (0.992, 1.005)  24-35 months: 0.995 (0.986, 1.003)  36-47 months: 0.999998 (0.993, 1.007)  48-59 months: 0.994 (0.988, 1.001) |
|  | 3 | Male: (ref)  Female: 1.001 (0.998, 1.005) | 6-11 months: (ref)  12-23 months: 0.997 (0.991, 1.003)  24-35 months: **0.993 (0.986, 0.9997)***  36-47 months: 0.998 (0.991, 1.004)  48-59 months: 0.995 (0.989, 1.001) |
| Minimum temperature | 0 | Male: (ref)  Female: 0.909 (0.725, 1.140) | 6-11 months: (ref)  12-23 months: 0.931 (0.546, 1.584)  24-35 months: 1.109 (0.630, 1.952)  36-47 months: 1.020 (0.685, 1.518)  48-59 months: 0.997 (0.567, 1.752) |
|  | 1 | Male: (ref)  Female: 0.904 (0.683, 1.196) | 6-11 months: (ref)  12-23 months: 0.946 (0.531, 1.685)  24-35 months: 1.337 (0.707, 2.530)  36-47 months: 0.889 (0.519, 1.521)  48-59 months: 1.317 (0.710, 2.444) |
|  | 2 | Male: (ref)  Female: 0.957 (0.795, 1.153) | 6-11 months: (ref)  12-23 months: 1.074 (0.754, 1.530)  24-35 months: 1.262 (0.824, 1.933)  36-47 months: 1.041 (0.723, 1.498)  48-59 months: 1.322 (0.896, 1.952) |
|  | 3 | Male: (ref)  Female: 0.930 (0.790, 1.094) | 6-11 months: (ref)  12-23 months: 1.067 (0.795, 1.432)  24-35 months: 1.192 (0.850, 1.671)  36-47 months: 1.055 (0.781, 1.425)  48-59 months: 1.149 (0.835, 1.581) |
| Maximum temperature | 0 | Male: (ref)  Female: 0.932 (0.824, 1.056) | 6-11 months: (ref)  12-23 months: 0.922 (0.694, 1.225)  24-35 months: 1.017 (0.759, 1.363)  36-47 months: 0.985 (0.769, 1.262)  48-59 months: 0.963 (0.718, 1.291) |
|  | 1 | Male: (ref)  Female: 0.935 (0.800, 1.092) | 6-11 months: (ref)  12-23 months: 0.935 (0.690, 1.267)  24-35 months: 1.250 (0.856, 1.825)  36-47 months: 0.921 (0.674, 1.258)  48-59 months: 1.201 (0.864, 1.669) |
|  | 2 | Male: (ref)  Female: 0.976 (0.870, 1.095) | 6-11 months: (ref)  12-23 months: 1.038 (0.842, 1.279)  24-35 months: 1.162 (0.896, 1.506)  36-47 months: 1.007 (0.814, 1.245)  48-59 months: 1.182 (0.940, 1.485) |
|  | 3 | Male: (ref)  Female: 0.983 (0.898, 1.075) | 6-11 months: (ref)  12-23 months: 1.051 (0.892, 1.239)  24-35 months: 1.119 (0.920, 1.360)  36-47 months: 1.010 (0.858, 1.189)  48-59 months: 1.113 (0.929, 1.333) |
| Average temperature | 0 | Male: (ref)  Female: 0.898 (0.742, 1.086) | 6-11 months: (ref)  12-23 months: 0.896 (0.584, 1.373)  24-35 months: 1.053 (0.687, 1.613)  36-47 months: 0.991 (0.678, 1.450)  48-59 months: 0.959 (0.628, 1.464) |
|  | 1 | Male: (ref)  Female: 0.918 (0.749, 1.125) | 6-11 months: (ref)  12-23 months: 0.931 (0.621, 1.395)  24-35 months: 1.314 (0.810, 2.130)  36-47 months: 0.904 (0.602, 1.356)  48-59 months: 1.264 (0.816, 1.959) |
|  | 2 | Male: (ref)  Female: 0.969 (0.839, 1.118) | 6-11 months: (ref)  12-23 months: 1.051 (0.806, 1.370)  24-35 months: 1.204 (0.869, 1.668)  36-47 months: 1.017 (0.776, 1.335)  48-59 months: 1.236 (0.924, 1.654) |
|  | 3 | Male: (ref)  Female: 0.966 (0.859, 1.087) | 6-11 months: (ref)  12-23 months: 1.061 (0.858, 1.313)  24-35 months: 1.150 (0.895, 1.477)  36-47 months: 1.023 (0.825, 1.267)  48-59 months: 1.134 (0.898, 1.431) |

*p<0.05

1 Adjusted for Sex, Urban/rural, Mothers’ education, Age, Altitude, Household electricity, Floor/Roof/Wall material, Wealth index, Hemoglobin level adjusted for altitude, BMI, Slept under a mosquito bed net the previous night, Medication taken for fever, Given medication to prevent malaria in applicable year, Sex*Precipitation. Results presented for Sex*Exposure

2 Adjusted for Sex, Urban/rural, Mothers’ education, Age, Altitude, Household electricity, Floor/Roof/Wall material, Wealth index, Hemoglobin level adjusted for altitude, BMI, Slept under a mosquito bed net the previous night, Medication taken for fever, Given medication to prevent malaria in applicable year, Age*Precipitation. Results presented for Age*Exposure

3 Exposure variable was centered to reduce collinearity

Table 4.4. Adjusted multilevel logistic regression model associations for every lag of 2015 for every exposure variable with malaria prevalence diagnosed by RDT in children ages 6 to 59 months including adjustment for sex and age as potential effect modification.

| Exposure variable | Lag | Model 1  OR (95% CI)^1^ | Model 2  OR (95% CI)^2^ |
| --- | --- | --- | --- |
| Precipitation | 0 | Male: (ref)  Female: 0.999 (0.995, 1.002) | 6-11 months: (ref)  12-23 months: 1.006 (0.999, 1.012)  24-35 months: 1.005 (0.998, 1.011)  36-47 months: 1.005 (0.999, 1.012)  48-59 months: 1.004 (0.997, 1.011) |
|  | 1 | Male: (ref)  Female: 0.999 (0.997, 1.001) | 6-11 months: (ref)  12-23 months: 1.002 (0.9996, 1.005)  24-35 months: 1.002 (0.999, 1.005)  36-47 months: **1.003 (1.000, 1.006)***  48-59 months: 1.003 (0.9995, 1.006) |
|  | 2 | Male: (ref)  Female: 0.999 (0.997, 1.001) | 6-11 months: (ref)  12-23 months: 1.002 (0.998, 1.006)  24-35 months: 0.999 (0.995, 1.003)  36-47 months: 1.002 (0.998, 1.006)  48-59 months: 1.001 (0.997, 1.005) |
|  | 3 | Male: (ref)  Female: 1.002 (0.999, 1.005) | 6-11 months: (ref)  12-23 months: 0.999 (0.996, 1.003)  24-35 months: 0.997 (0.992, 1.002)  36-47 months: 1.002 (0.999, 1.005)  48-59 months: 1.001 (0.997, 1.005) |
| Minimum temperature | 0 | Male: (ref)  Female: 0.958 (0.869, 1.057) | 6-11 months: (ref)  12-23 months: 1.070 (0.929, 1.232)  24-35 months: 1.087 (0.903, 1.309)  36-47 months: 1.065 (0.915, 1.241)  48-59 months: 1.023 (0.867, 1.208) |
|  | 1 | Male: (ref)  Female: 1.208 (0.668, 2.182) | 6-11 months: (ref)  12-23 months: **0.784 (0.616, 0.999)***  24-35 months: 0.802 (0.619, 1.040)  36-47 months: **0.741 (0.576, 0.953)***  48-59 months: **0.783 (0.615, 0.998)*** |
|  | 2 | Male: (ref)  Female: 1.032 (0.784, 1.360) | 6-11 months: (ref)  12-23 months: **0.635 (0.411, 0.981)***  24-35 months: 0.681 (0.425, 1.091)  36-47 months: **0.532 (0.340, 0.833)***  48-59 months: **0.598 (0.389, 0.919)*** |
|  | 3 | Male: (ref)  Female: 0.897 (0.700, 1.151) | 6-11 months: (ref)  12-23 months: **0.725 (0.529, 0.993)***  24-35 months: 0.813 (0.548, 1.206)  36-47 months: **0.693 (0.519, 0.925)***  48-59 months: **0.693 (0.489, 0.981)*** |
| Maximum temperature | 0 | Male: (ref)  Female: 1.072 (0.951, 1.208) | 6-11 months: (ref)  12-23 months: **0.782 (0.641, 0.956)***  24-35 months: 0.832 (0.667, 1.039)  36-47 months: 0.813 (0.660, 1.000)  48-59 months: 0.816 (0.658, 1.012) |
|  | 1 | Male: (ref)  Female: 1.041 (0.961, 1.128) | 6-11 months: (ref)  12-23 months: 0.884 (0.778, 1.005)  24-35 months: 0.906 (0.795, 1.033)  36-47 months: **0.867 (0.760, 0.991)***  48-59 months: 0.892 (0.787, 1.011) |
|  | 2 | Male: (ref)  Female: 1.040 (0.872, 1.241) | 6-11 months: (ref)  12-23 months: 0.774 (0.578, 1.037)  24-35 months: 0.835 (0.627, 1.113)  36-47 months: **0.688 (0.495, 0.955)***  48-59 months: 0.777 (0.577, 1.048) |
|  | 3 | Male: (ref)  Female: 0.922 (0.797, 1.067) | 6-11 months: (ref)  12-23 months: 0.886 (0.698, 1.125)  24-35 months: 1.012 (0.755, 1.356)  36-47 months: 0.911 (0.738, 1.126)  48-59 months: 0.894 (0.691, 1.156) |
| Average temperature | 0 | Male: (ref)  Female: 1.003 (0.839, 1.198) | 6-11 months: (ref)  12-23 months: 0.853 (0.678, 1.075)  24-35 months: 0.942 (0.663, 1.338)  36-47 months: 0.882 (0.700, 1.111)  48-59 months: 0.827 (0.620, 1.101) |
|  | 1 | Male: (ref)  Female: 1.050 (0.944, 1.167) | 6-11 months: (ref)  12-23 months: 0.847 (0.715, 1.003)  24-35 months: 0.870 (0.730, 1.036)  36-47 months: **0.821 (0.688, 0.979)***  48-59 months: 0.853 (0.722, 1.007) |
|  | 2 | Male: (ref)  Female: 1.041 (0.836, 1.297) | 6-11 months: (ref)  12-23 months: 0.708 (0.494, 1.013)  24-35 months: 0.767 (0.534, 1.104)  36-47 months: **0.610 (0.414, 0.900)***  48-59 months: 0.696 (0.484, 1.001) |
|  | 3 | Male: (ref)  Female: 0.902 (0.742, 1.097) | 6-11 months: (ref)  12-23 months: 0.813 (0.617, 1.073)  24-35 months: 0.942 (0.667, 1.330)  36-47 months: 0.819 (0.639, 1.051)  48-59 months: 0.806 (0.593, 1.095) |

*p<0.05

1 Adjusted for Sex, Urban/rural, Mothers’ education, Age, Altitude, Household electricity, Floor/Roof/Wall material, Wealth index, Hemoglobin level adjusted for altitude, Slept under a mosquito bed net the previous night, Medication taken for fever, Given medication to prevent malaria in applicable year, Has dwelling been sprayed in last 12 months?, Sex*Precipitation. Results presented for Sex*Exposure

2 Adjusted for Sex, Urban/rural, Mothers’ education, Age, Altitude, Household electricity, Floor/Roof/Wall material, Wealth index, Hemoglobin level adjusted for altitude, Slept under a mosquito bed net the previous night, Medication taken for fever, Given medication to prevent malaria in applicable year, Has dwelling been sprayed in last 12 months?, Age*Precipitation. Results presented for Age*Exposure

Table 4.5. Adjusted multilevel logistic regression model associations for every lag of 2012/13 for every exposure variable with malaria prevalence diagnosed by RDT in children ages 6 to 59 months including adjustment for sex and age as potential effect modification.

| Exposure variable | Lag | Model 1  OR (95% CI)^1^ | Model 2  OR (95% CI)^2^ |
| --- | --- | --- | --- |
| Precipitation | 0 | Male: (ref)  Female: 0.980 (0.902, 1.063) | 6-11 months: (ref)  12-23 months: 1.043 (0.893, 1.218)  24-35 months: 1.097 (0.932, 1.291)  36-47 months: 1.058 (0.905, 1.237)  48-59 months: 1.034 (0.901, 1.187) |
|  | 1 | Male: (ref)  Female: 0.994 (0.986, 1.002) | 6-11 months: (ref)  12-23 months: 1.007 (0.989, 1.025)  24-35 months: 1.012 (0.993, 1.032)  36-47 months: 1.010 (0.992, 1.028)  48-59 months: 1.005 (0.989, 1.021) |
|  | 2 | Male: (ref)  Female: 0.998 (0.994, 1.001) | 6-11 months: (ref)  12-23 months: 1.008 (0.9997, 1.017)  24-35 months: **1.012 (1.003, 1.020)***  36-47 months: **1.011 (1.003, 1.020)***  48-59 months: **1.008 (1.001, 1.016)*** |
|  | 3 | Male: (ref)  Female: 0.999 (0.997, 1.002) | 6-11 months: (ref)  12-23 months: 1.005 (0.999, 1.011)  24-35 months: **1.008 (1.003, 1.014)***  36-47 months: **1.007 (1.002, 1.013)***  48-59 months: **1.007 (1.001, 1.012)*** |
| Minimum temperature | 0 | Male: (ref)  Female: 1.073 (0.958, 1.202) | 6-11 months: (ref)  12-23 months: 1.089 (0.864, 1.372)  24-35 months: 1.116 (0.891, 1.398)  36-47 months: 1.103 (0.888, 1.369)  48-59 months: 1.101 (0.900, 1.348) |
|  | 1 | Male: (ref)  Female: 1.089 (0.999, 1.186) | 6-11 months: (ref)  12-23 months: 1.112 (0.931, 1.329)  24-35 months: 1.169 (0.981, 1.393)  36-47 months: **1.224 (1.035, 1.448)***  48-59 months: **1.205 (1.011, 1.436)*** |
|  | 2 | Male: (ref)  Female: 1.193 (0.99997, 1.423) | 6-11 months: (ref)  12-23 months: 0.778 (0.510, 1.188)  24-35 months: 0.835 (0.548, 1.273)  36-47 months: 0.852 (0.570, 1.273)  48-59 months: 1.005 (0.674, 1.497) |
|  | 3 | Male: (ref)  Female: 1.182 (0.922, 1.514) | 6-11 months: (ref)  12-23 months: 0.524 (0.265, 1.038)  24-35 months: 0.702 (0.360, 1.367)  36-47 months: 0.558 (0.298, 1.044)  48-59 months: 0.778 (0.408, 1.486) |
| Maximum temperature | 0 | Male: (ref)  Female: 1.064 (0.940, 1.204) | 6-11 months: (ref)  12-23 months: 1.145 (0.896, 1.462)  24-35 months: 1.238 (0.985, 1.555)  36-47 months: 1.163 (0.923, 1.465)  48-59 months: 1.190 (0.959, 1.475) |
|  | 1 | Male: (ref)  Female: **1.094 (1.013, 1.181)*** | 6-11 months: (ref)  12-23 months: 1.055 (0.900, 1.235)  24-35 months: 1.094 (0.937, 1.277)  36-47 months: 1.140 (0.979, 1.327)  48-59 months: 1.150 (0.987, 1.341) |
|  | 2 | Male: (ref)  Female: 1.092 (0.976, 1.222) | 6-11 months: (ref)  12-23 months: 0.832 (0.655, 1.057)  24-35 months: 0.793 (0.625, 1.006)  36-47 months: 0.840 (0.670, 1.054)  48-59 months: 0.925 (0.749, 1.144) |
|  | 3 | Male: (ref)  Female: 0.965 (0.860, 1.082) | 6-11 months: (ref)  12-23 months: 0.825 (0.657, 1.037)  24-35 months: **0.762 (0.609, 0.953)***  36-47 months: **0.753 (0.607, 0.934)***  48-59 months: **0.796 (0.643, 0.985)*** |
| Average temperature | 0 | Male: (ref)  Female: 1.075 (0.950, 1.217) | 6-11 months: (ref)  12-23 months: 1.122 (0.876, 1.437)  24-35 months: 1.185 (0.937, 1.500)  36-47 months: 1.140 (0.904, 1.437)  48-59 months: 1.152 (0.930, 1.427) |
|  | 1 | Male: (ref)  Female: **1.094 (1.008, 1.188)*** | 6-11 months: (ref)  12-23 months: 1.084 (0.914, 1.284)  24-35 months: 1.132 (0.958, 1.338)  36-47 months: **1.184 (1.007, 1.392)***  48-59 months: 1.180 (0.999, 1.394) |
|  | 2 | Male: (ref)  Female: 1.143 (0.990, 1.319) | 6-11 months: (ref)  12-23 months: 0.785 (0.569, 1.083)  24-35 months: 0.770 (0.560, 1.060)  36-47 months: 0.814 (0.601, 1.102)  48-59 months: 0.934 (0.699, 1.247) |
|  | 3 | Male: (ref)  Female: 0.995 (0.836, 1.184) | 6-11 months: (ref)  12-23 months: 0.684 (0.462, 1.011)  24-35 months: **0.659 (0.448, 0.967)***  36-47 months: **0.617 (0.428, 0.890)***  48-59 months: 0.711 (0.495, 1.021) |

*p<0.05

1 Adjusted for Sex, Urban/rural, Mothers’ education, Age, Altitude, Household electricity, Floor/Roof/Wall material, Wealth index, Hemoglobin level adjusted for altitude, BMI, Slept under a mosquito bed net the previous night, Medication taken for fever, Has dwelling been sprayed in last 12 months?, Sex*Precipitation. Results presented for Sex*Exposure

2 Adjusted for Sex, Urban/rural, Mothers’ education, Age, Altitude, Household electricity, Floor/Roof/Wall material, Wealth index, Hemoglobin level adjusted for altitude, BMI, Slept under a mosquito bed net the previous night, Medication taken for fever, Has dwelling been sprayed in last 12 months?, Age*Precipitation. Results presented for Age*Exposure
